# Transcriptional activity and strain-specific history of mouse pseudogenes

**DOI:** 10.1038/s41467-020-17157-w

**Published:** 2020-07-29

**Authors:** Cristina Sisu, Paul Muir, Adam Frankish, Ian Fiddes, Mark Diekhans, David Thybert, Duncan T. Odom, Paul Flicek, Thomas M. Keane, Tim Hubbard, Jennifer Harrow, Mark Gerstein

**Affiliations:** 1 Program in Computational Biology and Bioinformatics, Yale University, New Haven, CT 06520 USA; 2 Department of Molecular Biophysics and Biochemistry, Yale University, New Haven, CT 06520 USA; 3 Department of Life Sciences, Brunel University London, London, UB8 3PH UK; 4 Department of Molecular, Cellular & Developmental Biology, Yale University, New Haven, CT 06520 USA; 5 Systems Biology Institute, Yale University, West Haven, CT 06516 USA; 6 European Molecular Biology Laboratory, European Bioinformatics Institute, Wellcome Genome Campus, Hinxton, Cambridge CB10 1SD UK; 7 UC Santa Cruz Genomics Institute, University of California, Santa Cruz, CA 95064 USA; 8 Earlham Institute, Norwich Research Park, Norwich, NR4 7UH UK; 9 University of Cambridge, Cancer Research UK Cambridge Institute, Robinson Way, Cambridge, CB2 0RE UK; 10 Wellcome Trust Sanger Institute, Wellcome Trust Genome Campus, Hinxton, Cambridge CB10 1SA UK; 11 Department of Medical and Molecular Genetics, King’s College London, London, SE1 9RT UK; 12 Elexir, Wellcome Trust Genome Campus, Hinxton, Cambridge CB10 1SD UK; 13 Department of Computer Science, Yale University, New Haven, CT 06520 USA; 14 Department of Statistics & Data Science, Yale University, New Haven, CT 06520 USA

**Keywords:** Data integration, Genome informatics, Mouse, Genome evolution

## Abstract

Pseudogenes are ideal markers of genome remodelling. In turn, the mouse is an ideal platform for studying them, particularly with the recent availability of strain-sequencing and transcriptional data. Here, combining both manual curation and automatic pipelines, we present a genome-wide annotation of the pseudogenes in the mouse reference genome and 18 inbred mouse strains (available via the mouse.pseudogene.org resource). We also annotate 165 unitary pseudogenes in mouse, and 303, in human. The overall pseudogene repertoire in mouse is similar to that in human in terms of size, biotype distribution, and family composition (e.g. with GAPDH and ribosomal proteins being the largest families). Notable differences arise in the pseudogene age distribution, with multiple retro-transpositional bursts in mouse evolutionary history and only one in human. Furthermore, in each strain about a fifth of all pseudogenes are unique, reflecting strain-specific evolution. Finally, we find that ~15% of the mouse pseudogenes are transcribed, and that highly transcribed parent genes tend to give rise to many processed pseudogenes.

## Abstract (web summary)

Pseudogenes are key markers of genome remodelling processes. Here the authors present genome-wide annotation of the pseudogenes in the mouse reference genome and 18 inbred mouse strains, update human pseudogene annotations, and characterise the transcription and evolution of mouse pseudogenes.

## Introduction

The house mouse (*Mus musculus*) is a widely studied model organism^1^, with the field of mouse genetics accounting for more than a century of studies towards understanding mammalian physiology and development^2,3^. Advances of the Mouse Genome Project^4,5^ towards completing the de novo assembly and gene annotation of a collection of closely related mouse strains, and the wide variety of developmental and transcriptional data available from the mouse ENCODE project, provide a unique opportunity to get an in-depth picture of the evolution and variation amongst these important mammalian model organisms.

Mice frequently have been used as a model organism for studying human diseases due to their experimental tractability and similarities in their genetic makeup with humans^6^. This has resulted in the development of mouse models of specific diseases and the generation of knockout mice to recapitulate phenotypes associated with loss-of-function (LOF) mutations observed in humans. In general, a LOF event is a mutation that results in a modified gene product that lacks the molecular function of the ancestral gene. The advent of high-throughput sequencing has led to the emergence of new windows into the relationship between genotype and phenotype among the human population. Current efforts to catalogue genetic variation among closely related mouse strains extend this paradigm.

**Fig. 1:**
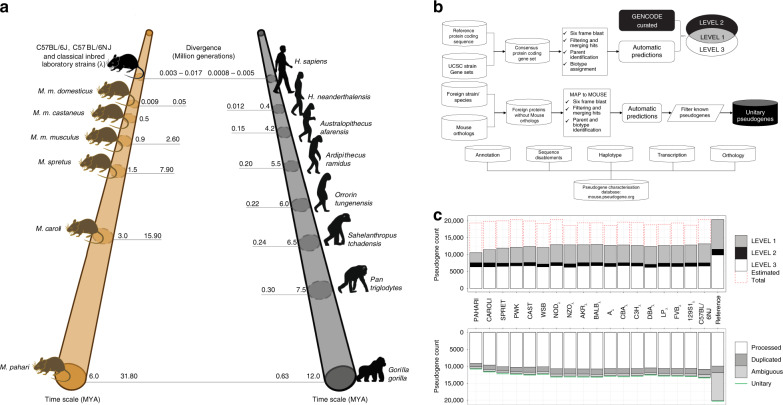
Pseudogene annotation. **a** Comparison on the evolutionary time scale of the divergence in selected primates and murine taxa. Each point on the primate time scale indicates the split from the human in million years (MYA). Each point on the murine time scale indicates the divergence time for splits among the wild-derived species and strains, and between *M. m. domesticus* and the classical laboratory inbred strains (denoted by λ). **b** (top) Pseudogene annotation workflow for mouse strains. **b** (middle) Unitary pseudogene annotation pipeline. **b** (bottom) Mouse pseudogene characterisation resource workflow. **c** Summary of mouse strains’ pseudogene annotation. Level 1 are pseudogenes identified by automatic pipelines and liftover of manual annotation from the reference genome; Level 2 are pseudogenes identified only through the liftover of manually annotated cases from the reference genome; Level 3 are pseudogenes identified only by the automatic annotation pipeline. The total number of pseudogenes in each biotype class and for each confidence level in each strain is available in Supplementary Table 5.

Human and mouse diverged around 90 million years ago (MYA)^7–9^. On the evolutionary scale, there is a larger range in divergence times amongst members of the genus *Mus* compared to those in genus *Homo* (Fig. 1a). Here, we investigate a number of mouse strains that have differences in their genetic makeup that manifest in an array of phenotypes, ranging from coat/eye colour to predisposition for various diseases^5^. Following an inbreeding process for at least 20 sequential generations, these mouse strains are homozygous at nearly all loci and show a high level of consistency at genomic and phenotypic levels^10^. This helps minimise a number of problems raised by the genetic variation between research animals^11^. The strain generation process also resulted in the fixation of variation between mouse strains, giving them unique genetic backgrounds with the potential to interact differently to an acquired or introduced mutation^12^. However, this process potentially introduced genetic contamination and intersubspecific introgression as revealed by the strains’ haplotype diversity^13,14^.

Often regarded as genomic relics, pseudogenes provide an excellent perspective on genome evolution^15,16^. In this work, we aim to provide a perspective on mouse evolutionary history by annotating and characterising the pseudogene complement. By definition, pseudogenes are DNA sequences that contain disabling mutations rendering them unable to produce a fully functional protein. Different classes of pseudogenes are distinguished based on their creation mechanism: (1) processed pseudogenes, formed through retrotransposition, (2) duplicated pseudogenes, formed through gene duplication and subsequent disablement of one of the duplicates, and (3) unitary pseudogenes formed when functional genes acquire disabling mutations that result in the inactivation of the original coding loci. Unitary pseudogenes are also characterised by the presence of a functional orthologous gene at the same locus in other species. In addition, there are loci that are present in a population both as a functional and a pseudogenised allele, with latter much more frequent. These are termed polymorphic pseudogenes^17^. Conversely, if the pseudogenised or disabled allele is rare, one usually refers to this as LOF event on a functional gene. Such pseudogenes represent disablements that have occurred on a more recent time scale. These are mutations that are not fixed in the population and are still subject to evolutionary pressures^17^. From a functional perspective, pseudogenes can be classified into three categories: ‘dead-on-arrival’, non-functional elements that are expected to be eliminated from the genome with time; ‘partially active’, exhibiting residual biochemical activity; and ‘exapted’ elements that have acquired new functions and can interfere with the regulation and activity of protein-coding genes.

Moreover, pseudogenes reflect changes in selective pressures and genome remodelling forces. Duplicated pseudogenes can reveal the history of gene duplication, one of the key mechanisms for establishing new gene functions^18^. While the majority of duplicated gene copies are eventually pseudogenised^19^, successfully retained paralogs can maintain a subset of the ancestral functions^20^ or acquire new functions^21^, processes known as sub- and neo-functionalisation, respectively^22^. Processed pseudogenes inform on the evolution of gene expression as well as the history of transposable element (TE) activity, whereas unitary pseudogenes are indicative of genes that died out. Thus, pseudogenes play an important role in evolutionary analysis representing multiple historical aspects of gene evolution, function, and activity, and in particular LOF events.

Unitary pseudogenes are an extreme case of LOF events, where mutations that result in complete inactivation of a gene are fixed in the population. In recent years, LOF mutations have become a key research topic in genomics. In general, a LOF event is detrimental to an organism’s fitness. However, a number of studies have showcased the evolutionary advantages of the accumulation and fixation of LOF mutations that result in the formation of unitary pseudogenes^23,24^.

Taken together, the well-defined evolutionary relationships between mouse strains and the wealth of associated functional data from the ENCODE project present an opportunity to investigate the processes underlying pseudogene biogenesis and activity. In this paper, we describe the pseudogene annotation and analysis of 18 widely used inbred mouse strains alongside the reference mouse genome. We provide the latest updates on the pseudogene annotation for both the mouse and human reference genomes, with a particular emphasis on the identification of new unitary pseudogenes. In addition, leveraging mouse developmental time-course RNA sequencing data, we explore whether pseudogene creation occurs primarily in the gametes or earlier in development in a germline precursor. Finally, we make all our data available online at mouse.pseudogene.org, a valuable resource for both population studies and the broader mouse genetics research community.

## Results

### Annotation

Using a combination of manual curation^25,26^ and automatic annotation with PseudoPipe^27^, we identified 20,397 pseudogenes in the mouse reference genome C57BL/6J (Fig. 1b, Supplementary Tables 1 and 2). The annotation files are available to download from mouse.pseudogene.org. The present dataset is a snapshot in an ongoing annotation process. As pseudogene assignments are highly dependent on the quality of the protein-coding annotation, the manually curated set provides a high-quality lower bound with respect to the true number of pseudogenes in the mouse genome, while the automatic annotation informs on the upper limit of the pseudogene complement size (Fig. 1c). In agreement with our previous work^25,26^, the manual and the automatic annotation show considerable overlap (over 83%) (Supplementary Table 1).

For human, we used a similar workflow to refine the reference pseudogene annotation to a high-quality set of 14,650 pseudogenes. The updated set contains considerable improvements in the characterisation of pseudogenes of previously unknown biotype (Supplementary Table 3). In both the human and mouse reference genomes, the majority of the annotations are processed pseudogenes, with a smaller fraction of duplicated pseudogenes.

Next, we focused on the annotation of pseudogenes in the mouse strains. The Mouse Genome Project has sequenced, assembled genomes, and developed a draft annotation for 12 classical laboratory mouse strains, three representatives of *Mus musculus* subspecies (*M. m. castaneus, M. m. musculus*, and *M. m. domesticus)*, and *Mus spretus*^28^. Another two distant species, *Mus caroli* and *Mus pahari*, were also sequenced and assembled^29^ (Supplementary Table 4).

Any analysis of the mouse strains should consider the highly inbred nature as well as accidental contamination that contributed to pervasive introgression of genomic sequences between strains^14^. Earlier studies have sought to explore the origins of classical laboratory strains by studying their haplotypes^13,14^. These analyses have shown that genomes of classical laboratory strains are a mosaic of regions derived to varying degrees from three *M. musculus* subspecies: (1) *M. m. domesticus* (94.3 ± 2.0%), (2) *M. m. musculus* (5.4 ± 1.9%), and (3) *M. m. castaneus* (0.3 ± 0.1%)^14^. A detailed summary of the genome composition for each strain is presented in Lilue et al.^28^ and Thybert et al.^29^.

Here, we developed an annotation and characterisation workflow for pseudogenes in mouse strains by leveraging the in-house automatic pipeline PseudoPipe and the set of manually curated pseudogenes from the mouse reference genome lifted over onto each strain (Fig. 1b). This combined identification process gives rise to three confidence levels reflecting the annotation quality: ‘Level 1’ includes pseudogenes with supporting evidence from both manual and automatic annotation, thus labelled as high-confidence entries; ‘Level 2’ includes annotations that are supported only by manually informed curation, and ‘Level 3’ includes pseudogenes identified by the automatic annotation pipeline alone. The level designation is in accord with common genome annotation practices^30^. Each identified pseudogene is associated with details about its transcript biotype, genomic location, structure, sequence disablements, and confidence level. A detailed overview of pseudogene annotation statistics including the number of pseudogenes, their confidence levels, and their biotypes is shown in Fig. 1c and Supplementary Table 5.

One challenge in developing a reliable annotation is identifying the optimal trade-off between the pipeline ‘specificity’ in producing highly accurate predictions, and ‘sensitivity’ in estimating the total number of pseudogenes in the genome. In developing a widely used annotation resource, we want as few false positive calls as possible. Conversely, when estimating the total number of pseudogenes in a given organism, we want a more balanced ratio of false positives to false negatives. Thus, as we aimed to produce annotation files for GENCODE that would serve as the gold standard in mouse pseudogene curation, we used as input only the conserved number of protein-coding genes between each analysed strain and the reference genome. Consequently, the present workflow was fine tuned to reduce the number of false positives by using a variety of filters (see ‘Methods’). However, this reduces the number of pseudogenes in distant species compared to that in the reference genome. This decrease is associated with the drop in the number of conserved input protein-coding genes (Supplementary Fig. 1a, b) and helps establish the lower bound of expected pseudogene complement size.

In addition, we estimated the total number of pseudogenes (see ‘Methods’ and Supplementary Table 5). The results suggest that all the studied strains have pseudogene complements of similar size. In terms of the pangenome nomenclature, we might say that we can annotate the conserved parts more accurately than the parts variable between species. The similarity in the total number of pseudogenes contrasts to the comparatively large levels of variation observed in the wild mice and relates to the origins of the classical inbred laboratory mice from a small number of founders^31^. Furthermore, previous studies in human and mouse have shown that even the natural levels of variation do not perturb the pseudogene numbers greatly. That is, there are not many polymorphic pseudogenes in either of these organisms^26^.

Currently, around 30% of pseudogenes in each strain are defined as Level 1, 10% are Level 2, and 60% are Level 3. The pseudogene biotype distribution across the strains closely follows the reference genome and is consistent with the biotype distributions observed in other mammalian genomes (e.g., human^25^ and macaque^26^) with ~80% processed pseudogenes and ~20% duplicated ones.

Next, we leveraged the information on ancestral haplotypes as defined by Yang et al.^14,32^ (see ‘Methods’) to characterise the pseudogenes by their origin. We lifted the imputed ancestral haplotypes from the reference mouse onto each of the assembled classical inbred strain genomes and intersected them with our pseudogene annotations. We annotated the most likely inferred ancestral haplotype for each pseudogene in the classical laboratory strains and made them available through our online resource at mouse.pseudogene.org. We found that for the pseudogenes conserved across multiple strains, the pseudogene origin pre-dated the differentiation of haplotypes, that is, on average many of the same conserved pseudogenes were given multiple haplotype designations in different strains (on average 3). Overall, this haplotype distribution suggests that only very few pseudogenes are spread and retained in new strains via introgression.

Finally, the density of pseudogene disablements exhibits a linear inverse correlation with the pseudogene age, as previously observed in the mouse reference genome and other mammals, with stop codons being the most frequent defect per base pair followed by deletions and insertions (Supplementary Fig. 2a, b).

Leveraging the available high-quality genome annotation in human, as well as our newly annotated mouse strains, we looked at identifying and characterising unitary pseudogenes.

As discussed above, unitary pseudogenes are the result of a complex interplay between LOF events and changes in evolutionary pressures. Thus, their importance resides not only in their ability to mark LOF events but also in their potential to highlight changes in the selective pressures in genome evolution. Unitary pseudogenes are formed through the inactivation of normal functional protein-coding genes. Thus, unitary pseudogenes do not have a functional counterpart in the same organism, and their identification is highly dependent on the quality of the reference genome, requiring a large degree of attention during the annotation process. Furthermore, unitaries are fundamentally defined relative to their orthologous functional protein-coding elements in another species.

**Fig. 2:**
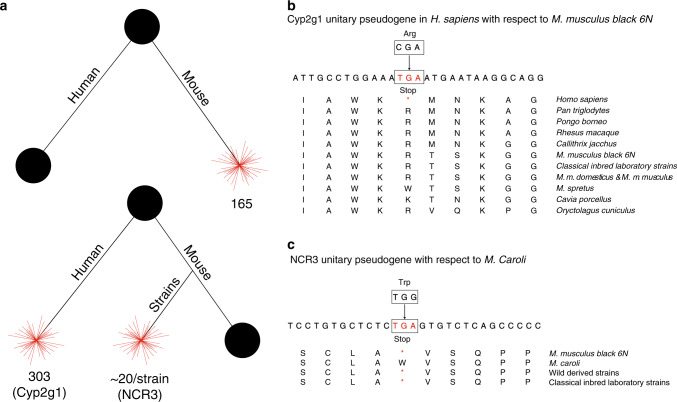
Unitary pseudogenes in human and mouse. **a** Summary of unitary pseudogenes with respect to human and mouse. The top panel shows the number of pseudogenes created in mouse with functional orthologs in human. The bottom panel shows the average number of pseudogenes that are present in 18 mouse strains and in the human genome with functional orthologs in mouse. The black disc indicates the presence of the functional protein coding gene, while the red star represents the pseudogene. **b** *Cyp2g1* LOF in human. **c** *NCR3* GOF mutation in *M. caroli* as compared to the reference genome and the other mouse strains.

Using a specialised unitary pseudogene annotation workflow (Fig. 1b) we identified 88 and 131 new unitary pseudogenes in human and mouse, respectively (Fig. 2a). These results bring the total number of unitaries in mouse to 165 and raise the size of unitary class in human to 303 entries (Supplementary Data 1). This is a considerable increase compared to previous GENCODE releases^17,30,33^ and can be largely attributed to improvements in mouse genome annotation and assembly. In mouse, the majority of the new unitary pseudogenes are associated with structural Zinc finger domains and immunoglobulin proteins (Supplementary Data 2). By contrast, in human, a large proportion of the new unitary pseudogenes are related to the chemosensory system (e.g., olfactory receptor proteins); reflecting the LOF events in these genes during the human lineage evolution. A known example of a human unitary pseudogene with functional counterparts in several mammals is the one associated with the *Cyp2g1* mouse protein (Fig. 2b). Here, the human gene acquired a C-to-T mutation equating to a stop codon in the middle of a coding exon, which resulted in gene disablement and thus the creation of a unitary pseudogene.

Furthermore, we found that new unitary pseudogenes are in general associated with lowly expressed protein-coding genes. In particular, more than half of the human functional orthologs that have been pseudogenised in mouse show no level of expression in the top-tier ENCODE cell lines (Supplementary Fig. 3a). The top broadly expressed protein-coding genes are Ribosomal proteins, NADH ubiquinone oxidoreductase, and the solute carrier *SLC25A6*. Similarly, a large fraction of the mouse functional orthologs of the human unitary pseudogenes shows low levels of expression across 18 mouse tissues (Supplementary Fig. 3b). Moreover, those that are transcribed are tissue specific.

The draft nature of the mouse strains’ annotation and assembly makes it difficult to identify unitary pseudogenes within them. On average, we found ~20 unitary pseudogenes in each strain (see ‘Methods’), with larger numbers for wild-derived inbred strains (Supplementary Data 3). We expect the actual number of unitary pseudogenes to depend on the evolutionary distance between strains. A comparison to unitary assignment in primates would be useful in this context—particularly, looking at whether or not the protein loss rate is comparable in both rodents and primates as it has been suggested by others^33^.

Improvements in the strain annotation will also allow us to annotate unitary pseudogenes in the reference with respect to the mouse strains. These elements will highlight not only reference genome LOF events, but also fixation of gain-of-function (GOF) mutations in divergent strains and species. This is the case for the *NCR3* gene, which shows an A-to-G GOF mutation in *M. caroli* reverting the initial TGA stop to a tryptophan codon, as compared to the pseudogenised counterparts in all the other mouse strains including the reference (Fig. 2c).

### Conservation and divergence

**Fig. 3:**
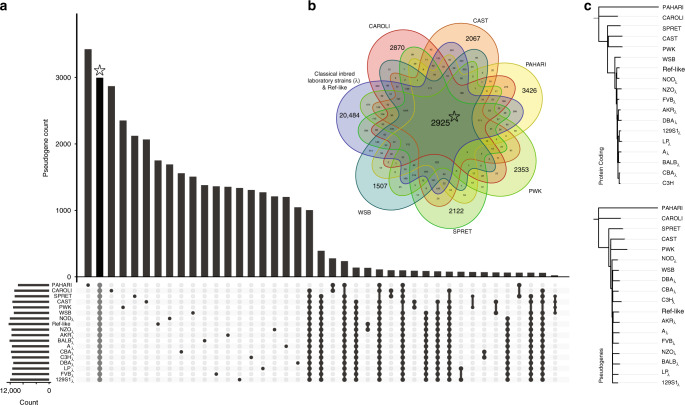
Pangenome distribution of pseudogenes. **a** Summary of pseudogene distribution in the pangenome mouse strain dataset. The classical laboratory inbred strains are listed in Supplementary Table 4, and the laboratory inbred ‘reference-like’ strain refers to C57BL/6NJ. The number of pseudogenes in each strain or group of strains is shown in corresponding Venn diagram intersections shown in (**b**). **b** 7-way Venn diagram of evolutionarily conserved and group-specific pseudogenes. **c** Phylogenetic trees for parents of evolutionarily conserved pseudogenes and evolutionary conserved pseudogenes. Bootstrap values are provided in mirror figure (Supplementary Fig. 3g).

To investigate the evolutionary history of pseudogenes in the mouse strains, we created a ‘pangenome’ pseudogene dataset containing 49,262 unique entries (Fig. 3a, b). We found 2,925 pseudogenes that are preserved across all strains. On average, each strain contains between 1,000 and 3,000 pseudogenes that do not have an orthologous counterpart in another species or strain, based on our imposed strict ortholog selection criteria (see ‘Methods’). By relaxing these constraints, we were able to estimate the minimum number of strain-specific pseudogenes. To this end, we identified a lower bound of on average 293 unique elements in each analysed genome. Moreover, the proportion of pseudogenes conserved only amongst outgroup *Mus* species, the wild-derived strains, or the classical inbred laboratory strains is considerably smaller than the number of pseudogenes conserved across all strains, suggesting that the bulk of the pseudogenes in each strain were created during the shared evolutionary history. In addition, the majority of pseudogenes have an ortholog in at least one other strain^14^.

To get a detailed picture of pseudogene origin and evolution, we clustered the strains based on the presence or absence of pseudogenes in syntenic regions. Using this binary information alone, we were able to recover known divergence patterns in the genus *Mus* showcasing pseudogenes as markers of genome evolution (Supplementary Fig. 3d–f). This approach however does not account for pseudogenes that have been lost after strain divergence, nor for the impact of introgression, which could lead to the introduction of a shared pseudogene not by descent. Thus, we next analysed the conservation patterns of pseudogenes across strains for any potential bias introduced by subspecies specific origin (see ‘Methods’). We observed no correlation between the percentage of a classical laboratory strain’s genome attributable to a given subspecies and the proportion of pseudogenes conserved between the strain and that subspecies’ representative strain (Supplementary Fig. 3c). This result suggests that the bulk of pseudogenes arose before these subspecies started diverging, and thus the subspecific origin or any potential cross contamination has no significant impact on the pseudogene annotation.

Next, we compared sequence divergence across the mouse strains. As pseudogenes evolve with little or no selective constraints^34^, they can provide a high-resolution picture of the evolutionary relationship between strains. To this end, we built a phylogenetic tree using ~1,500 pseudogenes conserved across all strains (see ‘Methods’, Fig. 3c, and Supplementary Fig. 3g). This pseudogene-based tree closely follows the tree constructed from protein-coding genes recovering the evolutionary relationships of the strains. This use of conserved syntenic pseudogenes for tree construction is particularly well suited for this analysis, as they are a set of high-confidence orthologous regions under less selective pressure than their coding counterparts.

### Genome evolution and plasticity

Taking advantage of available embryogenesis RNA-seq time-course data we studied pseudogene creation during early development^35^. Given that processed pseudogenes are formed through retrotransposition, we hypothesised that the expression level of the parent gene directly correlates with the number of processed pseudogenes^36^. Thus, we analysed the parent gene expression for developmental stages ranging from metaphase II oocytes to the inner cell mass. At every stage, the average expression level of parent genes is higher than that observed for non-parents. However, genes associated with large pseudogene families show low transcription levels during very early development, with high expression levels being achieved only at later stages. Furthermore, the correlation between the expression level of a gene and the number of pseudogenes associated with it improves as we progress through the developmental stages (Supplementary Fig. 4a, b). This suggests that pseudogenes are most likely generated by highly expressed housekeeping genes. By contrast, using germline data from Hammoud et al.^37^, we did not find any strong trends between parent gene expression and the number of pseudogenes, regardless of the spermatogenesis developmental stage (Supplementary Fig. 4c).

We further tested the correlation between high expression levels and the number of associated pseudogenes using RNA-seq data from adult mouse brain. Similar to our previous observations, the pseudogene parent genes show a statistically significant increase in average expression levels compared to non-pseudogene-generating protein-coding genes (Supplementary Fig. 4d, e).

**Fig. 4:**
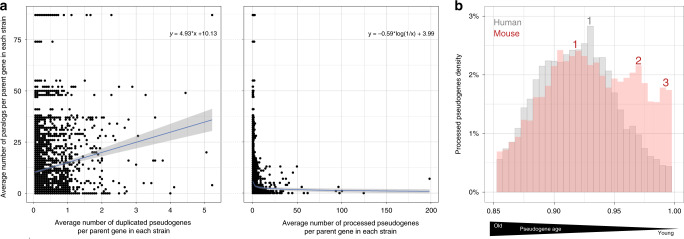
Pseudogene genesis. **a** Relationship between the number of pseudogenes and functional paralogs for a given parent gene (left—duplicated pseudogenes, right—processed pseudogenes). The number of parent genes associated with processed pseudogenes in strains is 11,571, and the number of parent genes associated with duplicated pseudogenes in strains is 3,758. The average number of pseudogenes per parent per strain was obtained by dividing the total number of pseudogenes across all strains by the total number of strains (18). Fitting lines show a vague correlation between the number of functional vs. disabled copies of a gene, with a linear fit for duplicated pseudogenes and a negative logarithmic fit for processed pseudogenes. The grey area is the ±SD (standard deviation) of the fitting line. **b** Distribution of reference processed pseudogenes (*y*-axis) in human (*n* = 8,081) and mouse (*n* = 9,979) as a function of age (*x*-axis). The pseudogene age is approximated as sequence similarity to the parent gene.

Next, we examined the degree to which the number of pseudogenes is related to the number of copies or functional paralogs of the parent gene (Fig. 4a). For duplicated pseudogenes, we observed a weak correlation between the number of paralogs and the number of pseudogenes of a particular parent gene. This result suggests that a highly duplicated protein family will tend to give rise to more disabled copies than a less duplicated family, if we assume that each duplication process can potentially give rise to either a pseudogene or a functional gene. This ratio of duplicated pseudogenes to paralogs in mouse is in accordance with previous observations in human^26,38^.

By contrast, for processed pseudogenes we observed a weak inverse correlation. This result implies that for large protein families we can expect to see a lower level of transcription for each family member, with high mRNA abundance potentially being achieved from multiple duplicated copies of a gene rather than increased expression of a single unit^39^. This is the case for the large PRAME (preferentially expressed antigen in melanoma) family, which have more than 80 paralogs in mouse and <2 processed pseudogenes per parent. These genes showed low or no expression in the studied adult brain (FPKM~0). On the opposite end of the spectrum, the GAPDH gene family, with seven paralogs and almost 200 processed pseudogenes, showed a significantly higher expression level (FPKM > 8) in the adult mouse brain (Supplementary Fig. 5a). Furthermore, we did not find any family-specific trends relating the number of paralogs and the number of pseudogenes (Supplementary Fig. 5b).

Next, we investigated the genomic mobile element content as well as the generation of processed pseudogenes on an evolutionary time scale (Fig. 4b and Supplementary Fig. 5c). This is a suitable follow up in our endeavour to understand the role of pseudogenes in genome evolution, as the majority of mouse and human pseudogenes are the result of retrotransposition processes mediated by short and long interspersed nuclear elements 1 (SINE and respectively LINE/L1).

In human, we observed a distribution of processed pseudogenes, with a single peak at 92.5% nucleotide sequence similarity to parents hinting at the burst of retrotransposition events that occurred 40 MYA at the dawn of the primate lineage^40,41^. By contrast, in mouse we found two successive peaks at 92.5 and 97% sequence similarity to parent genes. Moreover, in both human and mouse we observed a slight reduction in the number of newly created pseudogenes, which is likely a consequence of the stringent criteria used in calling pseudogenes at high sequence similarity to parents, and showcases the difficulty in annotating recent pseudogenization events. Overall, these results are supported by the large number of active TE in mouse, ~3,000 LINE/L1^42^ compared to just over 100 in human^43^, and reflect a continuous renewal of the processed pseudogene pool in mouse.

**Fig. 5:**
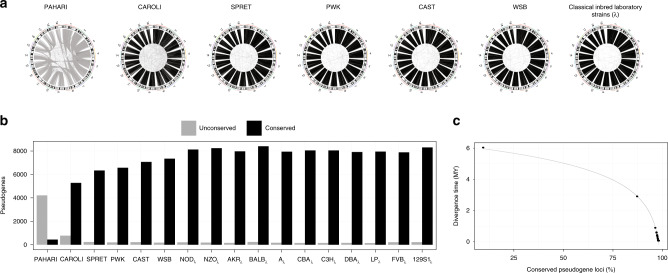
Pseudogene loci conservation across mouse strains. **a** CIRCOS-like plots showing the conservation of the pseudogene genomic loci between each mouse strain and the laboratory reference strain C57BL/6NJ. Grey lines indicate a change of the genomic locus between the two strains and connect two different genomic locations (e.g., a pseudogene located on chr7 in C57BL/6NJ and chr1 in *M. pahari*). Black lines indicate the conservation of the pseudogene locus. **b** The number of pseudogenes that are preserved or changed their loci between each strain/species and the laboratory reference strain. Associated data is available in Supplementary Table 6. **c** Strain speciation times as a function of percentage of conserved pseudogene loci between each strain/species and the laboratory reference, fitted by an inverse logarithmic curve.

The substantial proportion of strain- and group-specific pseudogenes, as well as the presence of active TE families, point towards multiple small-scale genomic rearrangements in the mouse genome evolution. To this end, we examined the preservation of genomic loci between each of the mouse strains and the reference genome for one-to-one pseudogene orthologs (Fig. 5a, b). We observed that on average more than 97.7% of loci are preserved across the classical laboratory strains, and 96.7% of loci are preserved with respect to the wild-derived inbred strains. By contrast, only 87% of *M. caroli* and 10% of *M. pahari* loci are preserved in the reference genome. The significant drop in the number of preserved pseudogene loci for these two strains is in agreement with the observed major karyotype-scale differences and large genomic rearrangements exhibited by *M. caroli* and *M. pahari*^29^. The proportion of non-preserved loci follows a logarithmic curve that matches closely the divergent evolutionary time scale of the mouse strains (Fig. 5c). Furthermore, looking at the pseudogene loci conservation as a function of biotype, we noticed that the deviation from the perfect fit is larger for non-processed pseudogenes compared to processed ones (Supplementary Fig. 6). This difference, however, can be attributed to the considerable differences in the input dataset size. Overall, the results suggest a uniform rate of genome remodelling processes across the murine taxa.

### Functional analysis

**Fig. 6:**
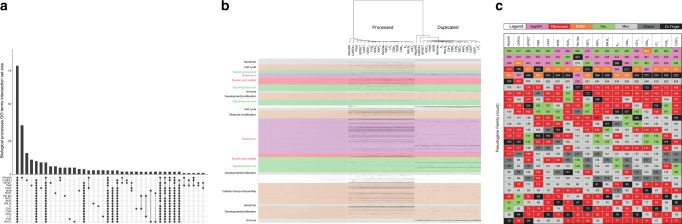
Functional analysis of pseudogenes. **a** Distribution of enriched GO biological processes terms across the mouse strains. Associated data is available in Supplementary Data 5. **b** Heatmap illustrating enrichment of GO biological processes terms across the mouse strains for the parent genes of processed and duplicated pseudogenes. GO terms (rows) are clustered by semantic similarity (colour). Each line in the heatmap indicates the presence of an enriched GO term associated with a strain’s pseudogene complement. The GO terms shown in colour indicate an association with the pseudogene family of similar colour in (**c**). **c** Summary of the top 24 Pfam pseudogene families in each mouse strain.

We integrated the annotations with gene ontology (GO) data in order to characterise the functions associated with pseudogene generation. We observed that the majority of top biological processes, molecular functions, and cellular component GO terms are shared across the strains (Fig. 6a). We also evaluated GO term enrichment among parent genes for both processed and duplicated pseudogenes across the mouse strains. Enriched GO terms were clustered based on semantic similarity and the strains were clustered based on GO term enrichment profile similarity. The resultant heatmap (Fig. 6b) enables us to identify both related terms with conserved enrichment across all strains as well as blocks of terms that exhibit conservation within a single or a few closely related strains. We observed a conserved enrichment for GO terms related to ribosomal functions, cell cycle, translation and RNA processing, and ubiquitination for processed pseudogenes. Among duplicated pseudogenes, we observed enrichment for apoptosis, sensory and smell processes, and immune functions. In addition, the GO terms that universally characterise the pseudogene complements in all the mouse strains are closely related to the family classification of pseudogenes. As we reported previously, the top pseudogene family is 7-Transmembrane^26^, reflecting the enrichment in olfactory receptors in the mouse. Similar to the human and primate counterparts, many top families in mouse pseudogenes are related to highly expressed and duplicated proteins such as GAPDH and Ribosomal proteins, and regulatory protein families such as the Zinc fingers (Fig. 6c)^44^.

A closer look suggests that the pseudogene repertoire also reflects individual strain-specific phenotypes (Supplementary Data 2). First, pseudogenes reflect duplication events linked with the emergence of an advantageous phenotype. This is the case for *M. spretus* genome, where we observed an enrichment of duplicated tumour repressor and apoptosis pathways genes^45^, and a corresponding increase in the number of associated pseudogenes. Second, we found pseudogenes reflecting the death of a gene family. As such, we observed an increase in the number of pseudogene-associated deleterious phenotypes. This is the case for the pseudogenisation of cytochrome c oxidase subunit VIa through accumulation of LOF mutations in the blind albino mouse strain, which is commonly linked with neurodegeneration^46^ and is characterised by brain lesions in the affected mice^10^.

Next, we focused our analysis on gene essentiality. Here, we found that essential genes (defined as required for an organismʼs survival^47^) are enriched in pseudogene parent genes. Specifically, they are approximately three times more abundant among parent genes (Supplementary Table 7). In general, essential genes are more highly transcribed than non-essential genes^48^, and thus might be associated with a higher propensity of generating processed pseudogenes. To this end, we evaluated the probability that a gene is essential by controlling for its transcription level and parent gene status (see ‘Methods’), and found that pseudogene parents are still 20% more likely to be essential genes compared to regular protein-coding genes (Supplementary Table 8).

We also looked to gain insight into the possible role of gene duplication in the enrichment of essential genes among the parent genes set by analysing the paralog content. In the reference mouse, 80.6% of non-essential genes and 74.1% of essential genes have paralogs. This is in agreement with previous work showing that non-essential genes are more likely than essential genes to be duplicated successfully^49^.

**Fig. 7:**
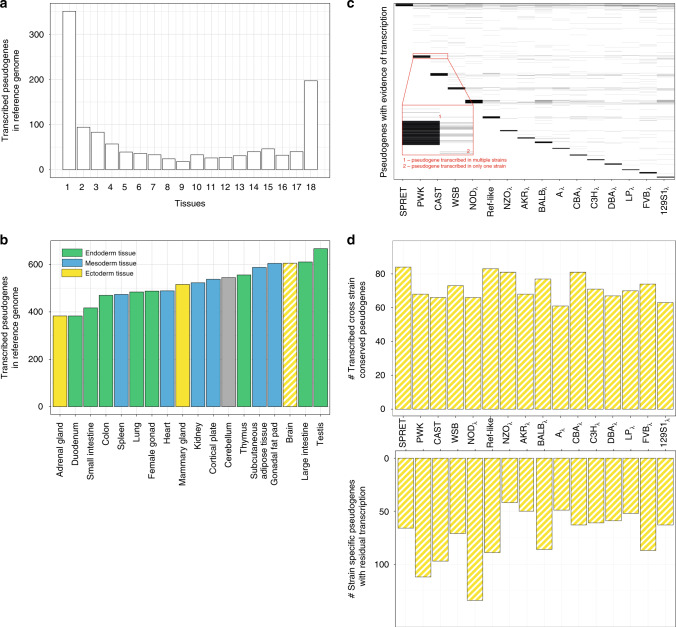
Pseudogene transcription and activity. **a** Cross-tissue pseudogene transcription in the mouse reference genome. The x-axis indicates the number of tissues in which a pseudogene is transcribed. **b** Distribution of pseudogene transcription in 18 adult mouse tissues. All data of the transcribed mouse reference genome pseudogenes in the 18 tissues is available in Supplementary Data 6. **c** Heatmap-like plot showing the distribution of transcribed pseudogenes (*y*-axis) in brain tissue for each wild-derived and classical laboratory mouse strain (*x*-axis). Each line corresponds to a transcribed pseudogene with an expression level higher than 2 (FPKM). When a line is present across multiple columns, it is indicative of a pseudogene expressed in all these strains. The dark bars at the top of each strain column are formed by multiple highly expressed pseudogenes. When a line is present in only one strain, and no other line is observed at the same level in any of the other strains, this suggests that the pseudogene expression is strain specific. **d** (top) Number of transcribed pseudogenes that are conserved across all the strains. **d** (bottom) Number of transcribed strain-specific pseudogenes in each mouse strain. Data recording the transcribed pseudogenes in brain for each strain is available from Supplementary Data 7.

Finally, we leveraged RNA-seq data from the Mouse Genome Project and ENCODE to study pseudogene transcriptional activity. This is thought to either relate to the exaptive functionality of pseudogenes or be a residual from their existence as genes. In both the human and mouse reference genomes, we found that about 15% of pseudogenes were transcribed across a variety of tissues, a result similar to previous pan-tissue analyses^25,26^ (Fig. 7a, b).

Due to restricted data availability, we focused our transcriptional analysis to a single tissue: adult brain from wild-derived and classical laboratory inbred strains. Overall, pseudogenes with strain-specific transcription were more common than those with cross-strain transcription (Fig. 7c, d). Moreover, the proportion of pseudogenes conserved across all strains that are expressed is constant (~2.5%) across the wild-derived and classical laboratory strains (Fig. 7d). By contrast, the fraction of transcribed strain-specific pseudogenes varies across the strains from 1.5 to 4% (Fig. 7d).

### Mouse pseudogene resource

We created a comprehensive resource that organises all of the pseudogene data across the available mouse strains and the reference genome, and is available at mouse.pseudogenes.org (Fig. 1b). The database contains information regarding strain and cross-strain annotation, pseudogene family and phenotypes, as well as pseudogene expression levels. All data are provided as flat files. Queries on specific pseudogenes will return the relevant annotation containing all pertinent associated information. The pseudogenes are labelled with a unique universal identifier as well as a strain-specific ID. This enables a pairwise comparison of pseudogenes between the various mouse strains and the investigation of similarities and differences between multiple strains of interest.

## Discussion

In this study, we report the updated and refined pseudogene annotation in the mouse and human reference genomes as part of the GENCODE project and describe the curation and comparative analysis of the first draft of pseudogene complements in 18 related mouse strains. By combining computational and manually informed annotations, we obtained a comprehensive view of the pseudogene content in genomes throughout the genus *Mus*. The current results represent a snapshot in an ongoing annotation process. Using information from manual curation and automatic annotation, we also computed an estimate of the total pseudogene complement size in each of the strains with a more balanced ratio of false positives to false negatives. Our previous experience with human genome annotation suggests that improvements in manual curation translate to a higher-quality pseudogene dataset^25,26^. Specifically, we expect the proportion of high confidence level pseudogene annotations (Level 1) to increase, consequently leading to a decrease in the number of pseudogenes identified only by computational methods (Level 3). We plan to update all of the annotations in line with major reference genome releases, which will ensure consistency in the nomenclature and characterisation of pseudogenes. This is particularly important for polymorphic and transcribed pseudogenes, as additional data could potentially allow reclassifications as protein-coding genes.

Comparable to our previous observations^26^, the pseudogene complement in mouse strains reflects an organism-specific evolution. Despite this, the pseudogenes share a number of similarities regarding their biogenesis and diversity. As such, we noticed a uniform ratio of processed-to-duplicated pseudogenes of 4 to 1 in all of the strains, a result consistent with previous observations in human^25,26^. The higher proportion of processed pseudogenes is in agreement with earlier findings that suggest that retrotransposition is the primary mechanism for pseudogene creation in numerous mammalian species^25^. Moreover, when we examined the TE activity, and in particular the L1 content, the genus *Mus* showed a sustained renewal of the pseudogene pool through multiple successive retrotransposition bursts. The sequence context of the processed pseudogenes indicates that the various retrotransposons exhibit differential contributions to the pseudogene content over time.

As a pseudogene’s likelihood of creation is related to its parent gene’s functional role and expression level, it can act as a record of its parent’s history. The link between the creation of processed pseudogenes from parent genes associated with key biological functions is further supported by an enrichment of parent genes among essential genes. Meanwhile, duplicated pseudogenes record events that shaped the genome environment and function during the organism’s evolution. As expected, we observed that parent genes have higher levels of expression relative to non-parents during both embryonic development and adulthood. Moreover, although time series expression analyses during embryonic development did not identify a single developmental time-point at which parent gene expression was strongly associated with pseudogenesis, a clear relationship was observed in the most mature cell types, suggesting that most pseudogenes’ creation is commonly related to the high expression levels of ubiquitous housekeeping genes.

By looking at the pangenome pseudogene repertoire, we distinguished three types of pseudogenes: universally conserved, multi-strain, and strain-specific, accounting for 6%, 23%, and 71% of the elements, respectively. Despite the large number of pseudogenes without an associated ortholog in the pangenome set, these account for only 20% of the total complement in any particular strain, a comparable proportion to the universally conserved pseudogenes in each strain. Moreover, by studying the conservation of their chromosomal location, we observed a stark contrast between the high level of genomic loci retention shared by the classical laboratory strains and the lack of conservation among the outgroup species hinting at multiple large-scale genomic rearrangements in genus *Mus*. This was especially noticeable in *M. pahari*, as was recently reported in a large-scale chromosomal imagining and karyotype analysis^29^.

Next, studying unitary pseudogenes and their functional orthologs allowed us to elucidate changing constraints and selective pressures in the genome evolution. We found that the enrichment of vomeronasal receptor unitary pseudogenes in human with respect to mouse highlights the loss of certain olfactory functions in humans.

Functional analysis showed an enrichment in housekeeping functions associated with conserved pseudogenes as illustrated by the presence of GAPDH, Ribosomal proteins, and Zinc finger nucleases as top Pfam families among the mouse pseudogenes, closely matching those seen in human. The GO enrichment analysis supports these results, with top terms including RNA processing and metabolic processes. In addition, we identified strain-specific functional annotations and suggested hypotheses as to what cellular processes and genes might underpin phenotypic differences between the mouse strains. For example, we observed that PWK/PhJ is associated with strain-specific GO terms for melanocyte-stimulating hormone receptor activity and melanoblast proliferation, which may play a role in the strain’s patchwork coat colour^50^. NZO/HlLtJ, an obesity prone mouse strain, was characterised by a specific enrichment in pseudogenes associated with defensin, a potential obesity biomarker^51^. Finally, examining the transcriptomic landscape, we observed evidence of both broadly and strain-specific expressed pseudogenes.

In summary, this comprehensive annotation and analysis of pseudogenes across 18 mouse strains provides support for conserved aspects of pseudogene biogenesis and expands our understanding of pseudogene evolution and activity. Integrating the annotations with existing functional data provided insight into the biological functions associated with pseudogenes and their parent genes. Furthermore, the well-defined relationships between the strains aided our evolutionary analysis of the pseudogene complements. Taken together, annotation of pseudogenes across a range of extensively used mouse strains and their integration into a comprehensive database with evolutionary and functional genomics data provide a useful resource for the broader research community and offers a comprehensive and rigorous background for future studies.

## Methods

### Datasets

Mouse reference genome is based on the *M. m*. strain C57BL/6J. The mouse reference annotation is based on GENCODE vM12/Ensembl 87.

The human reference genome annotation is based on GENCODE v25/Ensembl 87.

The 16 classical laboratory and wild-derived inbred strains’ (Supplementary Table 4) assemblies and strain-specific annotations were obtained from the Mouse Genome Project^28^ (http://www.sanger.ac.uk/science/data/mouse-genomes-project, last accessed on 21.08.2017). The classical laboratory strain C57BL/6NJ is a subline of the reference strain^10^. There is high sequence and evolutionary similarity between the reference genome single inbred strain C57BL/6J and the classical laboratory inbred mouse strain C57BL/6NJ. For the purpose of this study and in order to facilitate a reliable comparison across all the studied mouse genomes, we used the classical laboratory inbred strain C57BL/6NJ (labelled ‘reference-like’ or ‘ref-like’) as a reference point.

The two outgroup mouse species (Supplementary Table 4), *M. caroli* and *M. pahari*, were sequenced, assembled, and annotated in the protein-coding domain by Thybert et al.^29^.

### Divergence times in murine and primates

Human–primate lineage divergence and generation times were obtained from Langergraber et al.^52^. The divergence times for the wild-derived and classical laboratory strains were obtained from Vicens et al.^53^, Goios et al.^9^, and Zheng et al.^54^. The data for the two outgroup species’ divergence times was obtained from Thybert et al.^29^. The generation time for all the mice was estimated from the Mouse Genome Informatics database (MGI)^10^.

### Reference genome annotation

We manually curated 10,524 pseudogenes in the mouse reference genome (GENCODE M12) and 14,650 pseudogenes in the human reference genome (GENCODE v25). The manual annotation is based on the sequence homology to protein data from UniProt database^26^ and the protocol^25^ is summarised in Supplementary Fig. 7.

The number of manually annotated pseudogenes in the mouse strains is likely an underestimate of the true size of the mouse pseudogene complement given the similarities between the human and mouse genomes. Thus, to get a more accurate estimate of the number of pseudogenes in the mouse genome, we used a combination of two automatic annotation pipelines: PseudoPipe^27^ and RetroFinder^55^. PseudoPipe is the in-house comprehensive annotation pipeline that identifies and characterises pseudogenes based on their biotypes as either processed or duplicated. The automatic annotation workflow^25,26^ using PseudoPipe is summarised in Fig. 1b and focuses on a number of steps: (1) a six frame alignment of peptide sequences to the genome sequence using a translation blast approach, (2) filtering and merging of overlapping hits, (3) identification of potential pseudogene parents based on the sequence alignment score, and (4) assignment of pseudogenic biotype based on a number of features (e.g., conservation of intro-exon structure, presence of a PolyA sequence). PseudoPipe identified 22,811 mouse pseudogenes, of which 14,084 are present in autosomal chromosomes; this is comparable with previous reports in human (Supplementary Table 1). RetroFinder is a computational annotation pipeline focused on identifying retrotransposed genes and pseudogenes. Using RetroFinder, we were able to annotate 18,467 and 15,474 processed pseudogenes in mouse and human, respectively. There was good overlap between the two automatic identification pipelines with respect to the number of processed pseudogenes present in both organisms (Supplementary Table 1).

### Mouse strain annotation

The mouse strain pseudogene annotation workflow is summarised in Fig. 1b. The protein-coding input set contains the conserved protein-coding genes between each mouse strain and the reference genome. The number of shared transcripts follows an evolutionary trend with more distant strains having a smaller number of common protein-coding genes with the reference genome compared with more closely related classical laboratory strains. PseudoPipe was run with the strain conserved protein set as shown in Fig. 1b. Next, we used the HAL tools package^56^ to liftover the manually annotated pseudogenes from the mouse reference genome onto each strain using the UCSC multi-strain sequence alignments. We merged the two annotation sets using BEDTools^57^ with a 1 bp minimum overlap requirement. We extended the predicted boundaries to maximise the overlap and to ensure full annotation of the pseudogene transcript. Finally, we manually inspected the resultant annotation set in order to eliminate all potential false positives (e.g., pseudogene calls larger than 5 Kb or smaller than 100 bp, with poor protein-coding gene query similarity and coverage).

Next, we estimated the total number of pseudogenes in each strain by leveraging the close relationship between the mouse reference strain C57BL/6J and its related classical laboratory inbred strain, the ‘reference-like’ counterpart C57BL/6NJ. Thus, given the same genome assembly quality and protein-coding annotation, the two strains should exhibit a similar number of pseudogenes and protein-coding genes. However, the strains differ in the depth of their coding transcript annotations. This enables us to estimate the impact of differential annotation depth on the number of pseudogenes identified via the present workflow. Further, the differences between C57BL/6NJ and the reference genome will give an indication of the quality of the classical laboratory strains’ annotation. Consequently, we regarded C57BL/6NJ as a calibration strain.

To compute the number of pseudogenes in a particular strain based on the PseudoPipe annotation pipeline, we assumed that the correct number of pseudogenes is related to the number of input protein-coding transcripts. Moreover, observing that the number of conserved protein-coding transcripts between the mouse strains and the reference genome drops with increasing the evolutionary distance between the two, we worked on the premise that the actual total number of protein-coding gene transcripts should be constant across all mouse species. Thus, we defined a protein-coding transcript deflation factor as follows:

1 $$D\left( i \right) = \frac{{N\left( {T,i} \right)}}{{N\left( {T,ref} \right)}},$$

where *N*(*T*,*i)* is the number of protein-coding transcripts in strain *i* that are used as input, and *N*(*T*,*ref*) is the number of protein-coding transcripts in the reference genome.

Next, in order to get a realistic estimate of the total number of pseudogenes in each strain based on PseudoPipe annotations, we needed to correct for strain quality by considering how the deflation affects the number of pseudogenes in the calibration strain, as this number should be the same as the reference. We computed the calibration strain correction factor as follows:

2 $$C = \frac{{\frac{{N\left( {P,cal} \right)}}{{N\left( {P,ref} \right)}}}}{{\frac{{N\left( {T,cal} \right)}}{{N\left( {T,ref} \right)}}}},$$

where *N*(*P*,*cal*) is the number of PseudoPipe-annotated pseudogenes in the C57BL/6NJ calibration strain, *N*(*P*,*ref*) is the number of PseudoPipe pseudogenes annotated in the reference genome, *N*(*T*,*cal*) is the number of protein-coding transcripts used as input in the calibration strain, and *N*(*T*,*ref*) is the number of protein-coding transcripts used as input in the reference genome.

Thus, using the information from both the deflation factor and calibration strain correction factor we were able to estimate the number of pseudogenes in each strain based on the initial PseudoPipe pipeline output as follows:

3 $$M\left( {P,i} \right) = \frac{{N\left( {P,i} \right)}}{{D\left( i \right) \cdot C}},$$

where *N*(*P*,*i*) is the number of pseudogenes in strain *i*, *D*(*i*) is the deflation factor for strain *i*, and *C* is the calibration correction factor.

### Unitary pseudogene annotation pipeline

To get an overview of the unitary pseudogenes in each strain, we used the mouse reference genome as the required canonical organism and followed a similar workflow as described below. We modified PseudoPipe to allow cross-strain and cross-species protein-coding inputs. We annotated cross-organism pseudogenes as shown in Fig. 1b. We define ‘Functional organism’ as the genome providing the protein-coding information and thus containing a working copy of the element of interest; ‘Non-functional’ organism denotes the genome queried for unitary pseudogene presence. We used mouse reference peptides that are not present in the analysed strain, as input. The resulting dataset was subjected to a number of filters such as removal of previously known pseudogenes, removal of pseudogenes with parents that have orthologs in the annotated species, removal of pseudogenes that overlap with annotated protein-coding and ncRNAs loci, and removal of pseudogenes shorter than 100 bp. The filtered PseudoPipe set was intersected with the liftover of the protein-coding annotation from the functional organism using BEDTools^57^ with a minimum of 1 bp overlap required in order to validate the conservation of location and LOF of the protein-coding genes in the analysed strain/species. The intersection set was further refined by flagging protein-coding genes that have functional relatives (paralogs) in the non-functional organism. The remaining matches were subjected to manual inspection of the alignment.

### Pangenome dataset generation

We performed an ‘all-against-all’ liftover of pseudogene annotation using the HAL tools package and the UCSC multi-strain sequence alignment tool. Each liftover was intersected with the known strain annotation, and all of the entries that matched protein-coding genes or ncRNAs were removed. The resulting set was further filtered for conservation of pseudogene Ensembl ID (where available; Levels 1 and 2 pseudogenes), parent gene identity, pseudogene locus (reciprocal overlap of 90% or higher), pseudogene biotype, pseudogene length, and pseudogene structure.

Next, we integrated all filtered binary mappings in a master pan-strain set. The common entries were collapsed into a unique pangenome pseudogene reference. We obtained 49,262 pangenome pseudogenes. A total of 1,158 pangenome entries were multi-matching across strains.

The number of strain-specific pseudogenes was calculated as the difference between the total number of pseudogenes and the number of pseudogenes with at least one identified ortholog in another strain/subspecies. The high specificity and accuracy in annotating orthologs translates into high sensitivity in identifying strain-specific pseudogenes. Thus, the current number of strain-specific pseudogenes is an upper bound of the expected dataset size. To estimate the lower bound of the number of strain-specific pseudogenes, we relaxed the cut-off level in the conservation of pseudogene locus and sequence overlap (see Supplementary Fig. 8). The lower the threshold, the larger the number of called orthologs and, consequently, a smaller number of strain-specific pseudogenes. The minimum number of expected strain-specific pseudogenes in the current dataset was calculated under the hypothesis that a strain-specific pseudogene will have 0% sequence overlap with any annotated elements in any of the other strains. Thus, there is a minimum of 295 strain unique pseudogenes on average in any of the 18 mouse genomes.

### Phylogenetic analysis

Sequences of the 1,460 pseudogenes were randomly selected out of the total 2,925 conserved pseudogenes in the 18 mouse strains; this accounts for ~50% of the total number of conserved pseudogenes. The rationale for the use of a reduced pseudogene set was to reduce the computational demands of sequence alignment and phylogenetic tree generation. The use of pseudogenes and protein-coding genes across the genome reduces any potential bias introduced by the strains’ mosaicism by averaging out the contribution of any given genomic region. In addition, the use of pseudogenes conserved across all strains removes pseudogenes shared among subsets of strains due to contamination via introgression.

For each of the 18 mouse genomes, the extracted sequences were concatenated into a strain-specific contig (supergene) in the same order in which they occur in the various strains. The order of the pseudogene sequences was kept the same in all 18 contigs. Preserving the same order of pseudogenes or protein-coding genes across all strains eliminates any potential bias. Also, by selecting samples from the conserved pseudogenes set that are randomly distributed across the genome we are able to by-pass any potential bias introduced by the strain mosaicism. Thus, the resulting phylogeny depends only on sequence evolution. The 18 supergenes were subjected to multi-sequence alignment using MUSCLE aligner^58^ under standard conditions. Similarly, the sequences of parent protein-coding genes of the 1,460 pseudogenes were assembled into a strain-specific sequence and aligned using MUSCLE. The tree was generated with PhyML using the Tamura-Nei genetic distance model and simultaneous Nearest Neighbour Interchange build method with *M. pahari* as the outgroup^59^. For both the pseudogene and parent gene, tree alignment and tree construction were done at the nucleotide level.

### Cross-strain contamination and haplotype analysis

We computed the normalised number of pseudogenes conserved between each classical lab strain and the two wild-derived laboratory strains most representative of the subspecies with smaller contributions to the genomes of the classical lab strains (PWK/PhJ for *M. m. musculus* and CAST/EiJ for *M. m. castaneus*) as a function of the percentage of the classical lab strain’s genome derived from these two subspecies. Next, we calculated the correlation between the normalised number of conserved pseudogenes and the percentage of the classical strain’s genome attributable to the other *Mus* subspecies in either case and found no statistically significant level of correlation between the two factors.

### Associating pseudogene annotations with subspecific origin and inferred haplotype

We utilised genome annotations generated in Yang et al.^14^, which segmented the genomes of mouse strains into regions associated with different subspecies and inferred ancestral haplotypes based on data from the Mouse Diversity Array (MDA)^32^. These annotations were based on the mm9 mouse reference genome. We first used UCSC LiftOver to map the annotations into the mm10 reference genome and then further mapped the annotations into each strain genome using halLiftover. This process fragmented the annotations and we merged overlapping annotations with the same haplotype or subspecies in order to generate a more concise set, which was then intersected with the pseudogene annotations in each strain. Each pseudogene in each strain has been thus annotated with the associated inferred ancestral haplotype and the associated subspecies specific origin. The two annotation files are available for download from mouse.pseudogene.org.

### Genome mappability maps

We created mappability maps for the mouse reference genome and the 18 mouse strains using the GEM library^60^. The workflow is composed of indexing the genome using gem-indexer, followed by creation of the map using a window of 75 nucleotides under the following conditions: -m 0.02 -T 2.

### Parent gene expression analysis

RNA-seq mouse tissue data were obtained from ENCODE. The complete list of experiments used is available in Supplementary Data 4. We estimated the expression levels of the pseudogene parent protein-coding genes using a workflow involving the following steps: filtering the protein-coding genes for uniquely mappable regions longer than 100 bp, mapping reads using TopHat2^61^, selecting high-quality mapped reads with a quality score higher than 30 using samtools^62^, and calculating the expression of FPKM levels using Cufflinks^63^. Transcriptional activity of pseudogene parent genes during early embryonic development was investigated using RNA-seq data as processed and described in Wu et al.^35^. Raw sequencing data and processed data containing FPKM levels at each embryonic stage are available on the SRA under Series GSE66582 (https://www.ncbi.nlm.nih.gov/sra?term=SRP055882).

### Transposable elements analysis

TEs in human and mouse reference genomes were informed from the RepeatMasker library Repbase 21.11 and using RepeatMasker 3.2.8^64^. We extracted all four major groups of repeats, SINE, LINE, LTR, and DNA, and identified all the processed pseudogenes associated with L1 elements. Next, we binned the L1 annotated pseudogenes into age groups based on their sequence similarity to the parent gene, with younger elements exhibiting a higher sequence similarity and older elements showing a large sequence divergence when compared to the functional gene counterparts.

### Gene ontology and Pfam analysis

Linking of GO terms to the pseudogene parent genes was conducted using the R package biomaRt^65^. Visualisation of shared and distinct GO term sets among the strains was done using the R package UpSetR^66^. Enrichment of GO terms among the pseudogene parent genes and clustering of mouse strains based on similar enrichment profiles was performed using the goSTAG software package^67^. Semantic clustering of the GO terms was done with OntologyX packages^68^. Parent genes were labelled with both strain and biotype information in order to better evaluate differences in the pseudogene complements based on their mechanism of creation. Analysis of the Pfam representation in the pseudogene complements was performed by associating the pseudogene with the protein family of its parent gene^69^.

### Gene essentiality enrichment analysis

Lists of essential and non-essential genes were compiled using data from the MGI database and recent work from the International Mouse Phenotyping Consortium^70^. The non-essential gene set with Ensembl identifiers contained 4,736 genes compared to 3,263 essential genes.

In order to evaluate the impact of parent gene status on the probability of a gene being essential while controlling for transcription, we fit a linear probability model and a probit model for the probability that a gene is essential given its transcription level and parent gene status using the StatsModels package in Python. The linear probability model fits an ordinary least squares regression of gene essentiality on parent gene status and transcription level. Although the linear probability model generally estimates relationships well close to the mean of the independent variables, it often loses explanatory power at low and high values of these variables. Because of this deficiency, we also used the probit model, which is similar to the linear probability model but instead fits the data to a cumulative Gaussian distribution. Around the mean values, we found that parent gene status increases the probability of essentiality by around 20% in both models.

### Pseudogene transcription

We estimated the pseudogene transcription levels for the mouse reference in 18 adult tissues following a similar protocol as the one described earlier for calculating the expression of protein-coding genes. We have successfully used this method in the past^26^ using ENCODE RNA-seq data (Supplementary Data 4). The pseudogene sequences were filtered for uniquely mappable exon regions longer than 100 bp. Next the RNA-seq raw data was mapped using TopHat and the mapped reads were filtered for quality scores higher than 30. The resulting alignments were quantified using Cufflinks. A pseudogene was considered transcribed if it had an FPKM larger than 3.3 in accord with previous studies^26^.

RNA-seq data from mouse adult brain were obtained from the Mouse Genome Project for 12 classical laboratory and four wild-derived strains, available from ftp://ftp-mouse.sanger.ac.uk/REL-1509-Assembly-RNA-Seq, last accessed on August 8, 2017. The whole brain RNA-seq data are currently available at https://www.ebi.ac.uk/arrayexpress/experiments/E-MTAB-615/samples/, last accessed on May 28, 2020. Next, we created mappability maps for each of the 16 mouse strains’ genomes and selected only the pseudogene exons in uniquely mappable regions that were longer than 100 bp for further transcription analysis. The pseudogene transcription levels in mouse strains were estimated using a similar workflow as described above. The transcription cut-off level was set to 1.

### Mouse pseudogene resource

All of the annotation data produced in the analysis was collected and made available online through mouse.pseudogene.org (Fig. 1b). Pseudogene annotation information encompasses the genomic context of each pseudogene, its parent gene and transcript Ensembl IDs, the corresponding mouse reference pseudogene Ensembl ID, the level of confidence in the pseudogene as a function of agreement between manual and automated annotation pipelines, and the pseudogene biotype.

Information on the cross-strain comparison of pseudogenes is derived from the liftover of pseudogene annotations from one strain onto another and subsequent intersection with that strain’s native annotations. The database provides liftover annotations and information about intersections between the liftover and native annotations. Furthermore, homology information provides links between the well-characterised mouse strain collection.

Links between the annotated pseudogenes, their parent genes, and relevant functional and phenotypic information help inform biological relevance. In the database, the Ensembl ID associated with each parent gene is linked to the appropriate MGI gene symbol, which serves as a common identifier to connect to the phenotypic information. These datasets include information on gene essentiality, Pfam families, GO terms, and transcriptional activity.

### Reporting summary

Further information on research design is available in the Nature Research Reporting Summary linked to this article.

## Supplementary information

Supplementary Information

Reporting Summary

Description of Additional Supplementary Files

Supplementary Data 1

Supplementary Data 2

Supplementary Data 3

Supplementary Data 4

Supplementary Data 5

Supplementary Data 6

Supplementary Data 7

Supplementary Data 8

## Data Availability

All data generated and analysed in this work is available at http://mouse.pseudogene.org. The GENCODE manual annotation data used in this study is available at https://www.gencodegenes.org. The mouse strains’ assembled genomes from the Mouse Genome Project are available at https://www.sanger.ac.uk/science/data/mouse-genomes-project. Mouse tissue RNA-seq data are available at https://www.ebi.ac.uk/arrayexpress/experiments/E-MTAB-615/samples/. Mouse development RNA-seq data are available on the SRA under Series GSE66582 [https://www.ncbi.nlm.nih.gov/sra?term=SRP055882]. Pfam protein families are available at http://xfam.org.

## Code availability

The pseudogene annotation pipeline is freely available at http://pseudogene.org/pseudopipe.
