# Supplementary material for: Transcriptional activity and strain-specific history of mouse pseudogenes
Source: Nat Commun. 2020 Jul 29;11:3695. doi: 10.1038/s41467-020-17157-w (PMC7392758; doi:10.1038/s41467-020-17157-w)
Supplement: Supplementary file 1 — Supplementary Information [file 41467_2020_17157_MOESM1_ESM.pdf]

Supplementary Information

Transcriptional activity and strain-specific history of mouse pseudogenes

Sisu, Muir et al.

Supplementary Figures

A

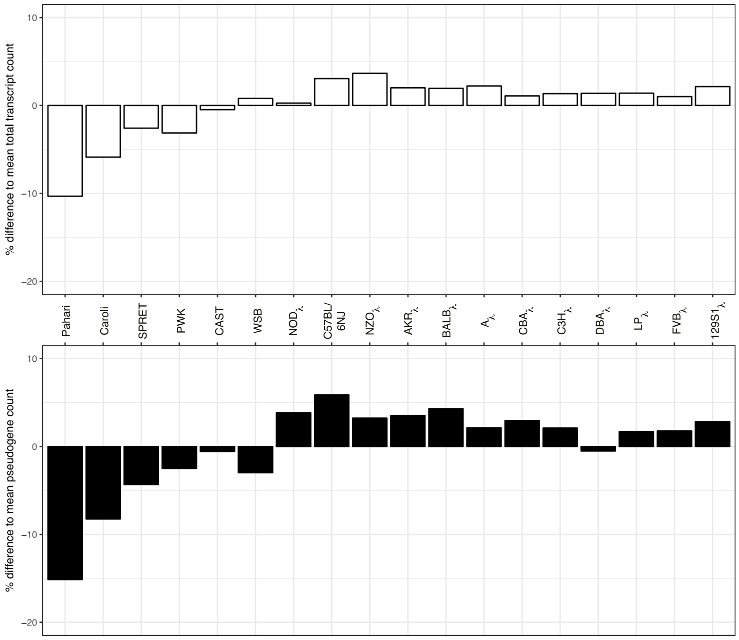

B

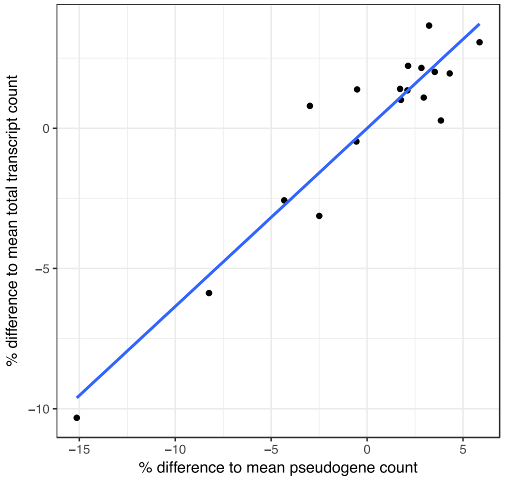

Supplementary Figure 1. A – The percentage difference between the number of pseudogene/conserved protein coding transcripts per strain and the average across all strains. Associated data is available from **Supplementary Data 7.** B – Scatterplot of the percentage difference between the number of pseudogene/conserved protein coding transcripts per strain and the average across all strains. Pearson correlation coefficient = 0.94.

A

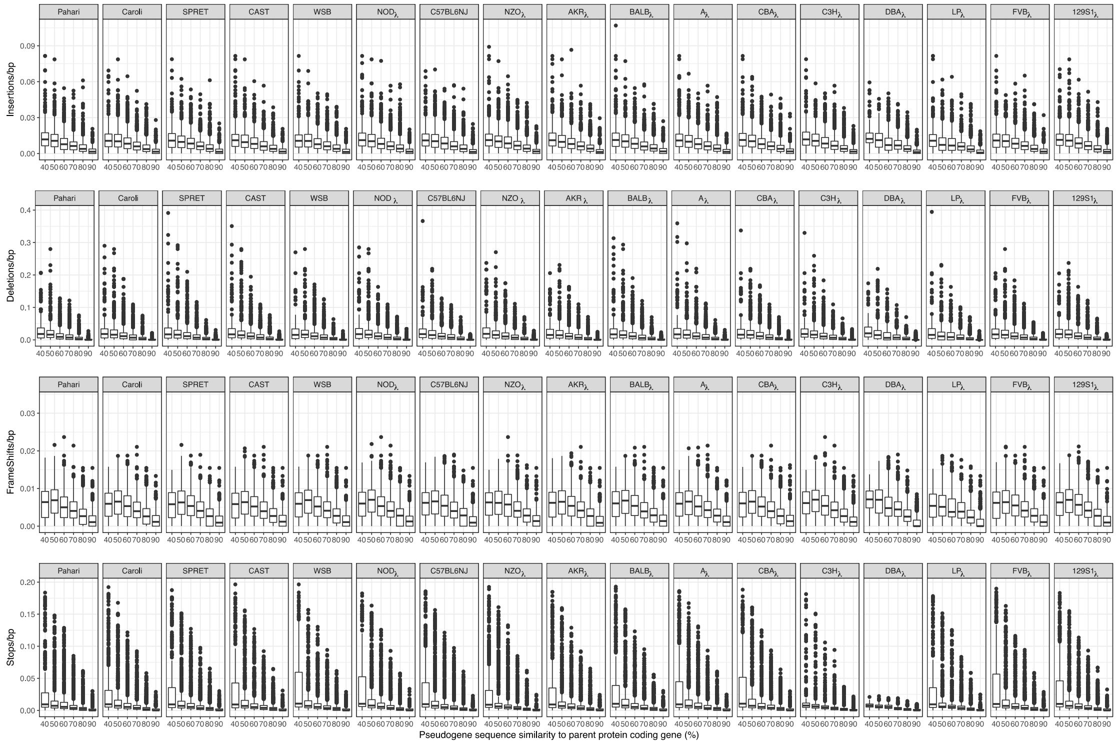

B

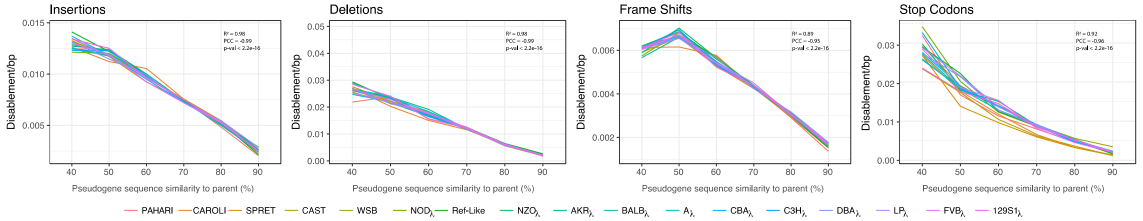

Supplementary Figure 2. A – Box plot distribution of pseudogene disablements per bp in 18 mouse strains. Centre line indicates the median value, box limits are the upper and lower quartiles, whiskers are 1.5x interquartile range, and the points are the outliers. Associated data is available from **Supplementary Data 7.** B – Trends of disablement density per bp as function pseudogene sequence similarity to the parent in 18 mouse strains. The R^2, and the Pearson correlation coefficient (PCC) are shown on the graph. The p-value was calculated using an ordinary ANOVA test.

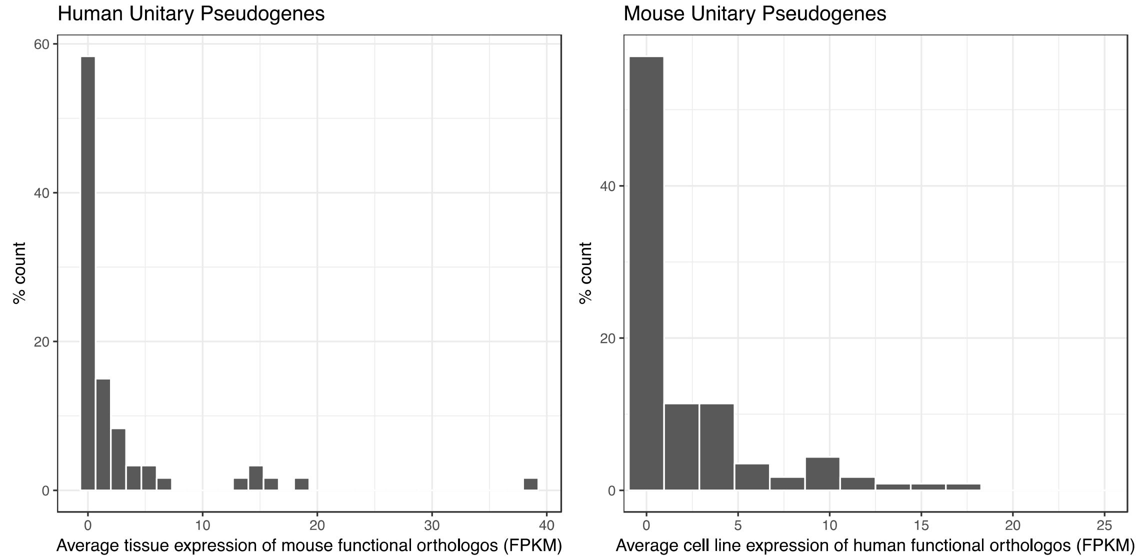

Supplementary Figure 3. A – Histogram distribution of expression levels for the functional paralogs of unitary pseudogenes. The left hand graph gives the average tissue (n=18) expression level for the mouse functional paralogs that are pseudogenised in human (n=60), while the right hand graph show the average ENCODE cell line (n=17) expression level for the human functional paralogs that are unitary pseudogenes in mouse (n=114).

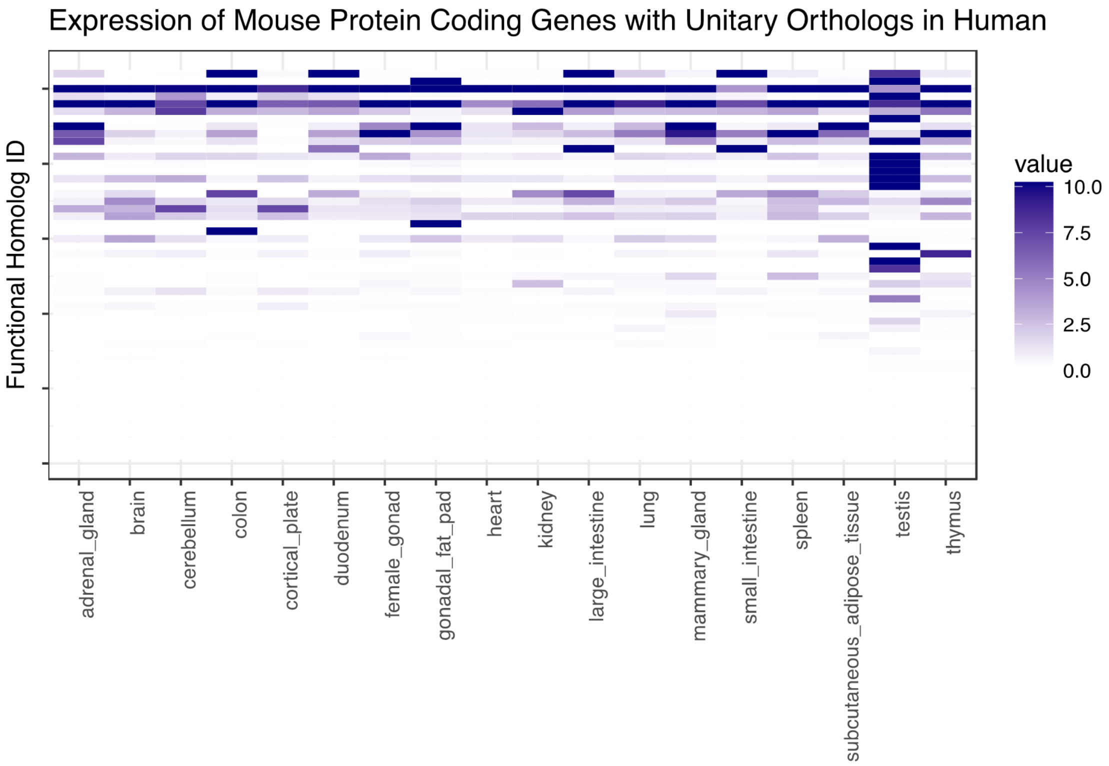

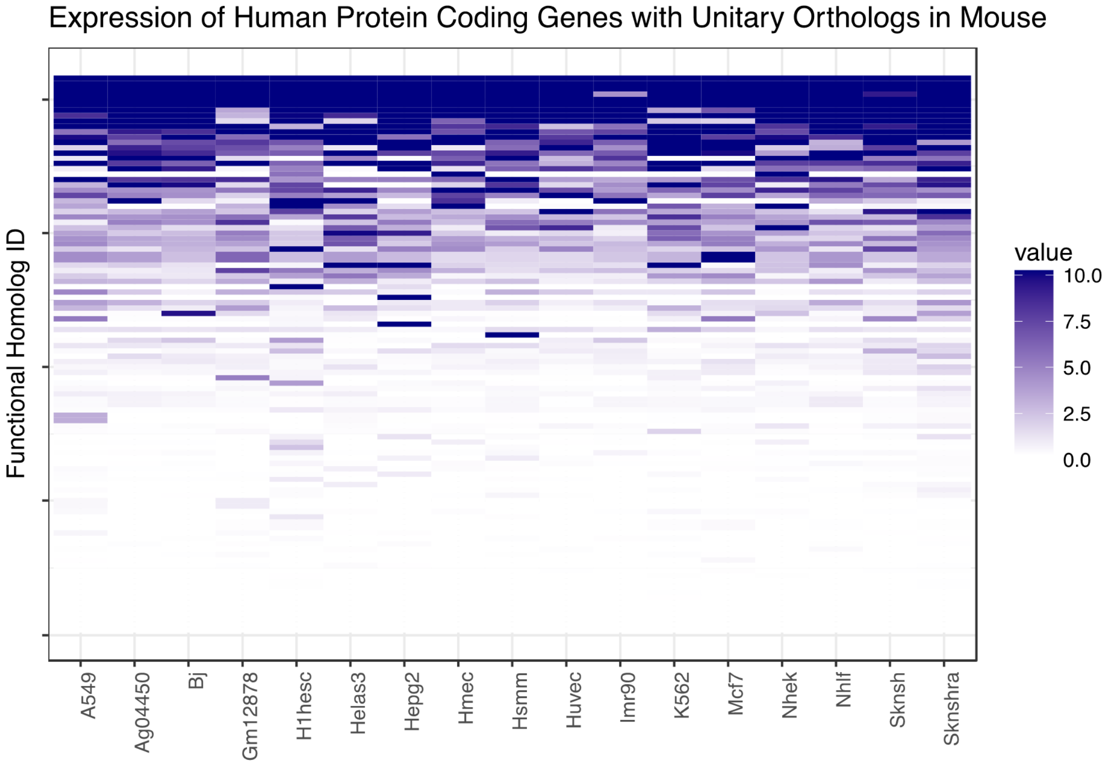

Supplementary Figure 3. B – Distribution of expression levels for the functional paralogs of unitary pseudogenes per tissue in mouse (top) and per ENCODE cell line in human(bottom). The colour scale top value corresponds to an expression score of greater or equal to 10 FPKM.

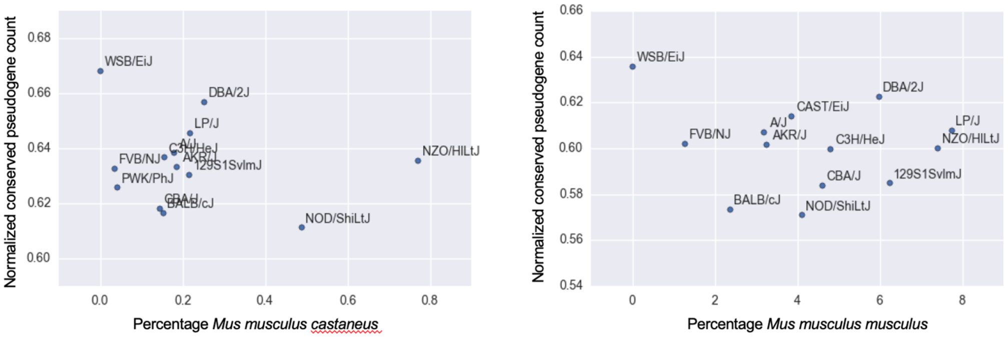

Supplementary Figure 3. C – Normalized number of pseudogenes shared between each classical laboratory inbred strain and the wild-derived strains representative of the two *M. musculus* subspecies from which smaller fractions of the classical lab strain genomes are derived (left: CAST/EiJ for *M. m. castaneus* and right: PWK/PhJ for *M. m. musculus)*.

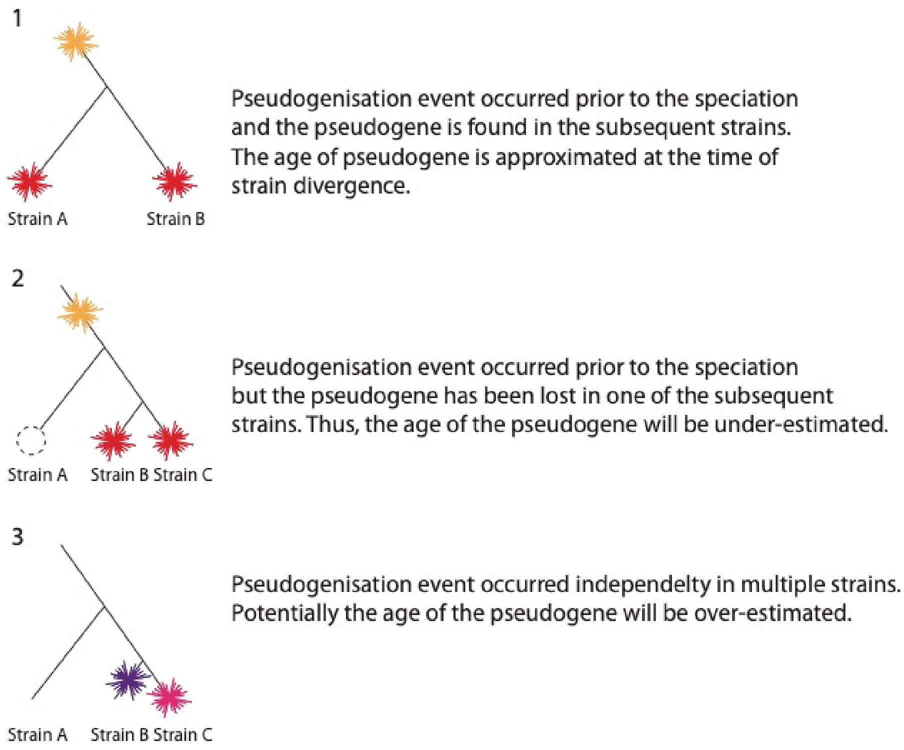

Supplementary Figure 3. D – Bias inducing events in estimating the age of pseudogene based on its presence or absence in various strains. The star shape indicates a pseudogenisation event. The dashed circle indicates the loss of the pseudogene in a strain.

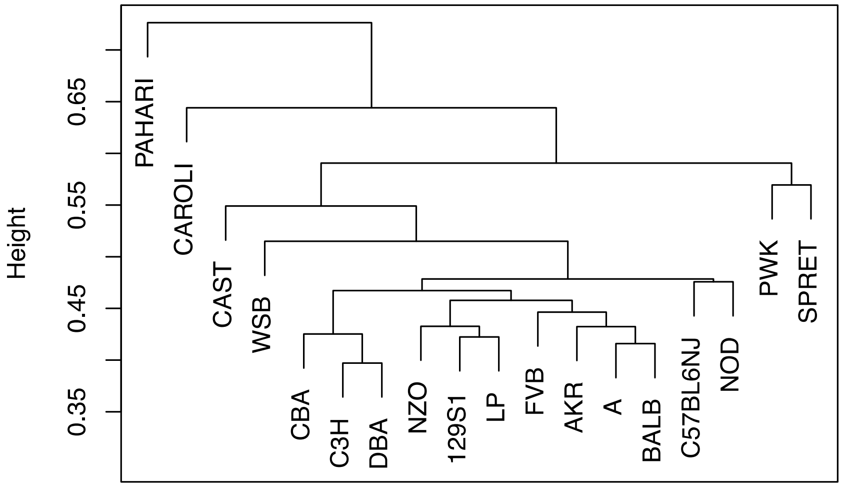

Supplementary Figure 3. E – Mouse lineage evolutionary tree based on the presence and absence of orthologous and strain specific pseudogenes across the strains using as input a binary matrix (1-pseudogene is present and 0 –the pseudogene is absent from the strain).

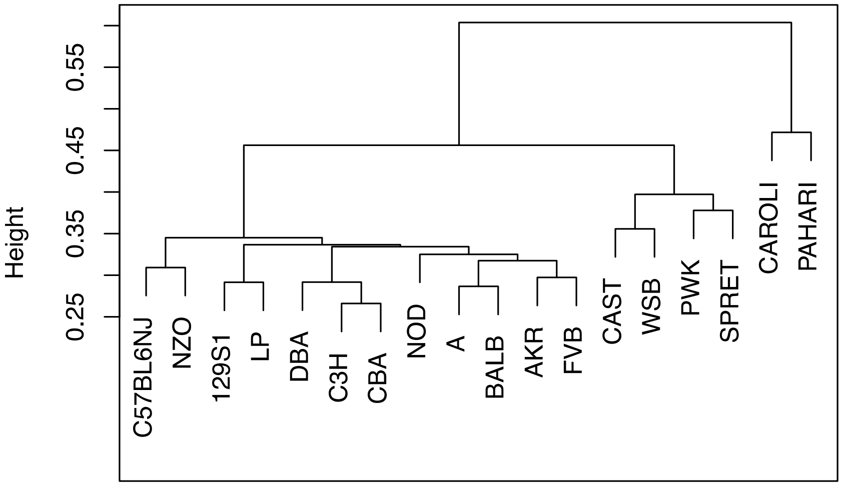

Supplementary Figure 3. F – Mouse lineage evolutionary tree based solely on the presence and absence of orthologous pseudogenes across the strains using as input a binary matrix (1-pseudogene is present and 0 –the pseudogene is absent from the strain).

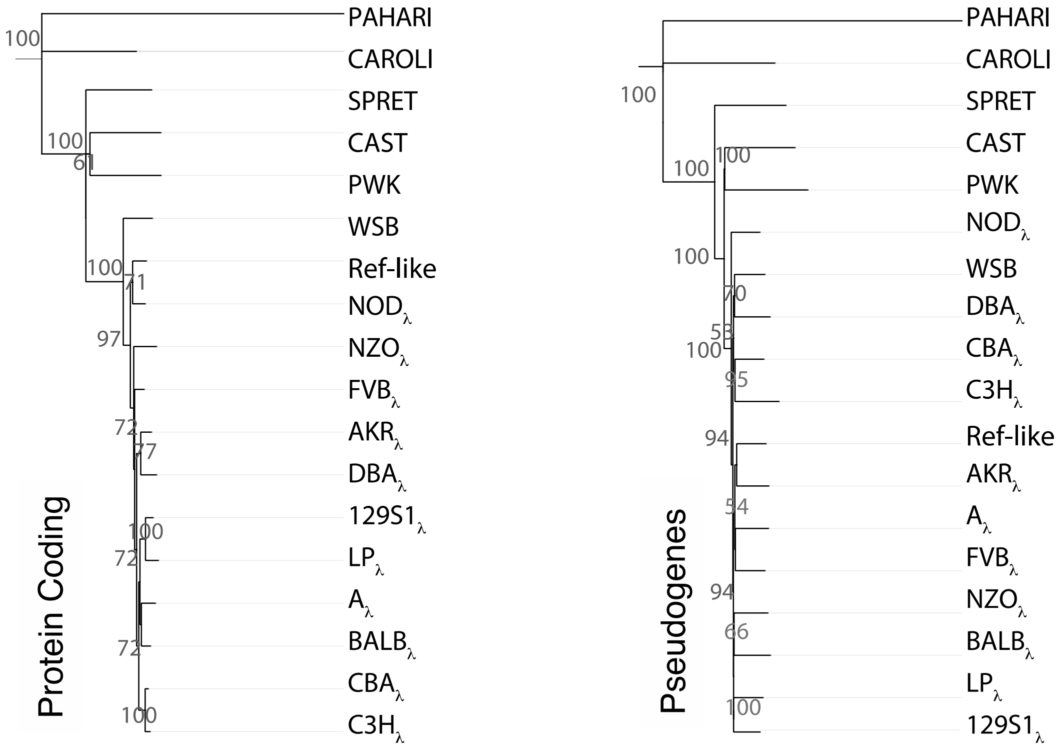

Supplementary Figure 3. G – Mirror of **Figure 3C** highlighting the phylogenetic trees of evolutionary conserved pseudogenes and pseudogenes parents with the associated bootstrap values on the branches.

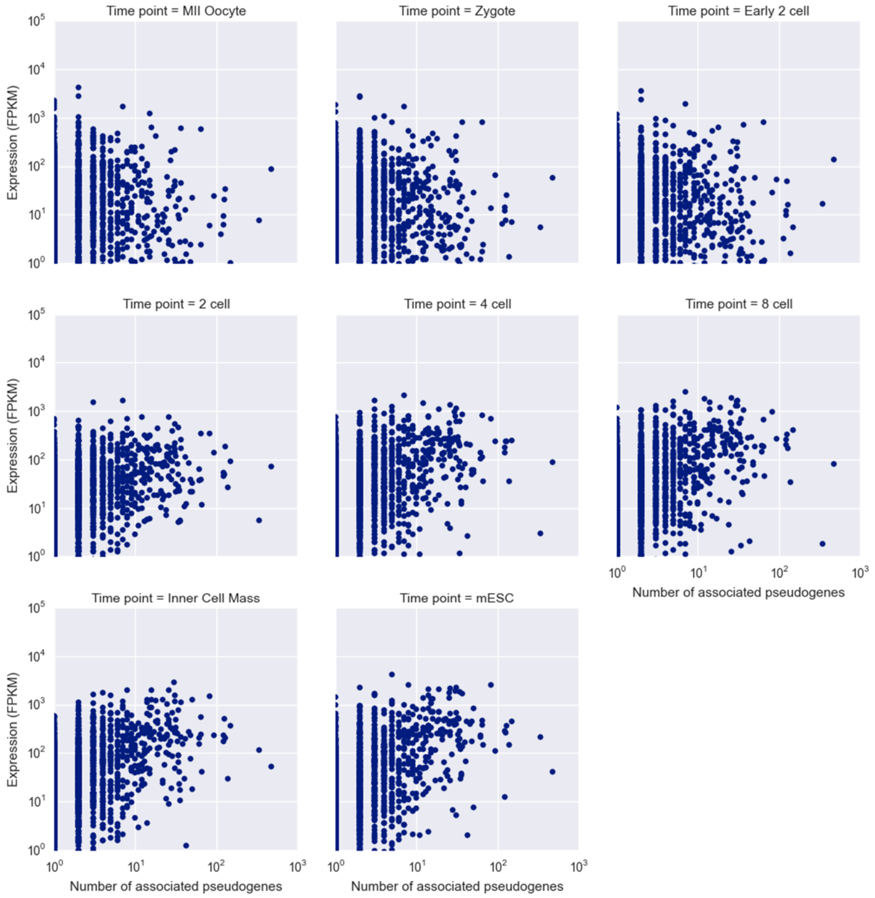

Supplementary Figure 4. A. Transcriptional activity of a gene vs the number of its associated pseudogenes at different early embryonic developmental time points.

All Genes (7,797)			
Embryonic Stage	Slope	R^2	P-Value
MII_oocyte	0.000680	.0004	0.0803
zygote	0.003195	0.0027	4.72e-06
early_2cell	0.003324	.0029	2.30e-06
2cell	0.016201	.0185	1.42e-33
4cell	0.013029	.0267	7.37e-48
8cell	0.011471	.0292	3.18e-52
ICM	0.012790	.0431	1.26e-76
mESC	0.012985	.0477	7.24e-85

Parent Genes (1,015)			
Embryonic Stage	Slope	R^2	P-Value
MII_oocyte	0.000195	0.000	0.930
zygote	0.003353	0.001	0.281
early_2cell	0.002932	0.001	0.298
2cell	0.011617	0.007	0.00634
4cell	0.011475	0.015	8.63e-05
8cell	0.010365	0.018	2.30e-05
ICM	0.016475	0.041	6.83e-11
mESC	0.015057	0.044	1.22e-11

Supplementary Figure 4. B. Regression statistics defining the transcriptional activity of a gene vs the number of its associated pseudogenes at different early embryonic developmental time points.

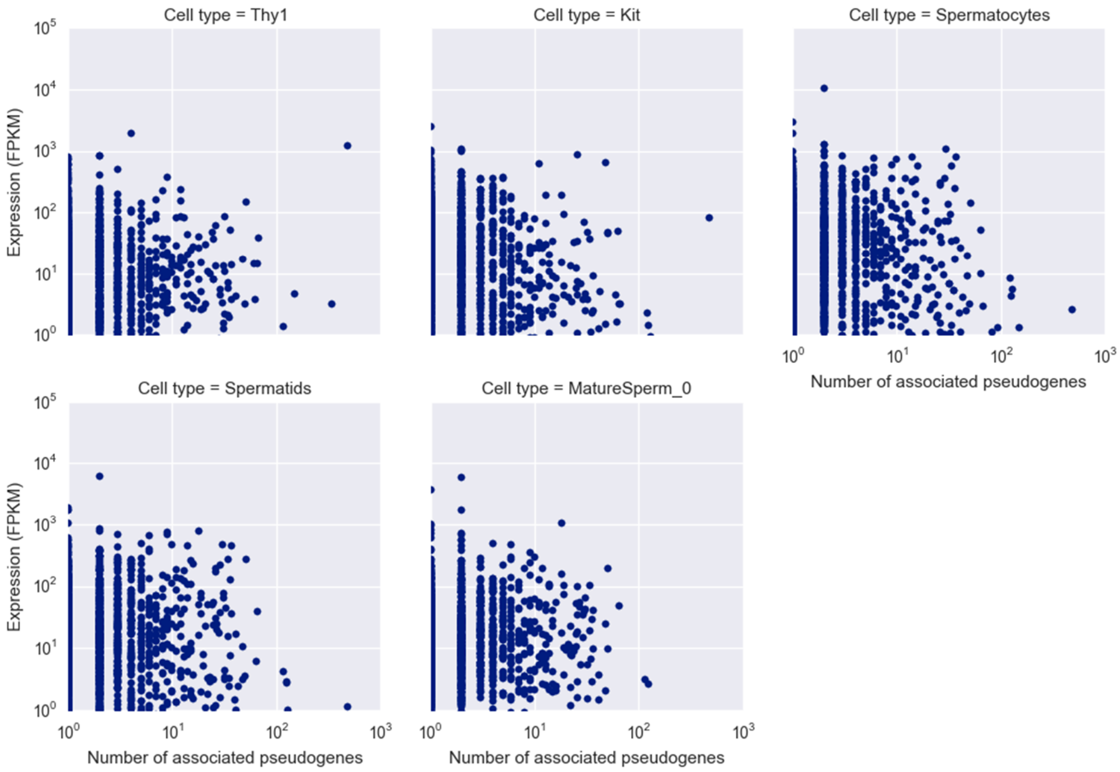

Supplementary Figure 4. C. Transcriptional activity of a gene vs the number of its associated pseudogenes during spermatogenesis.

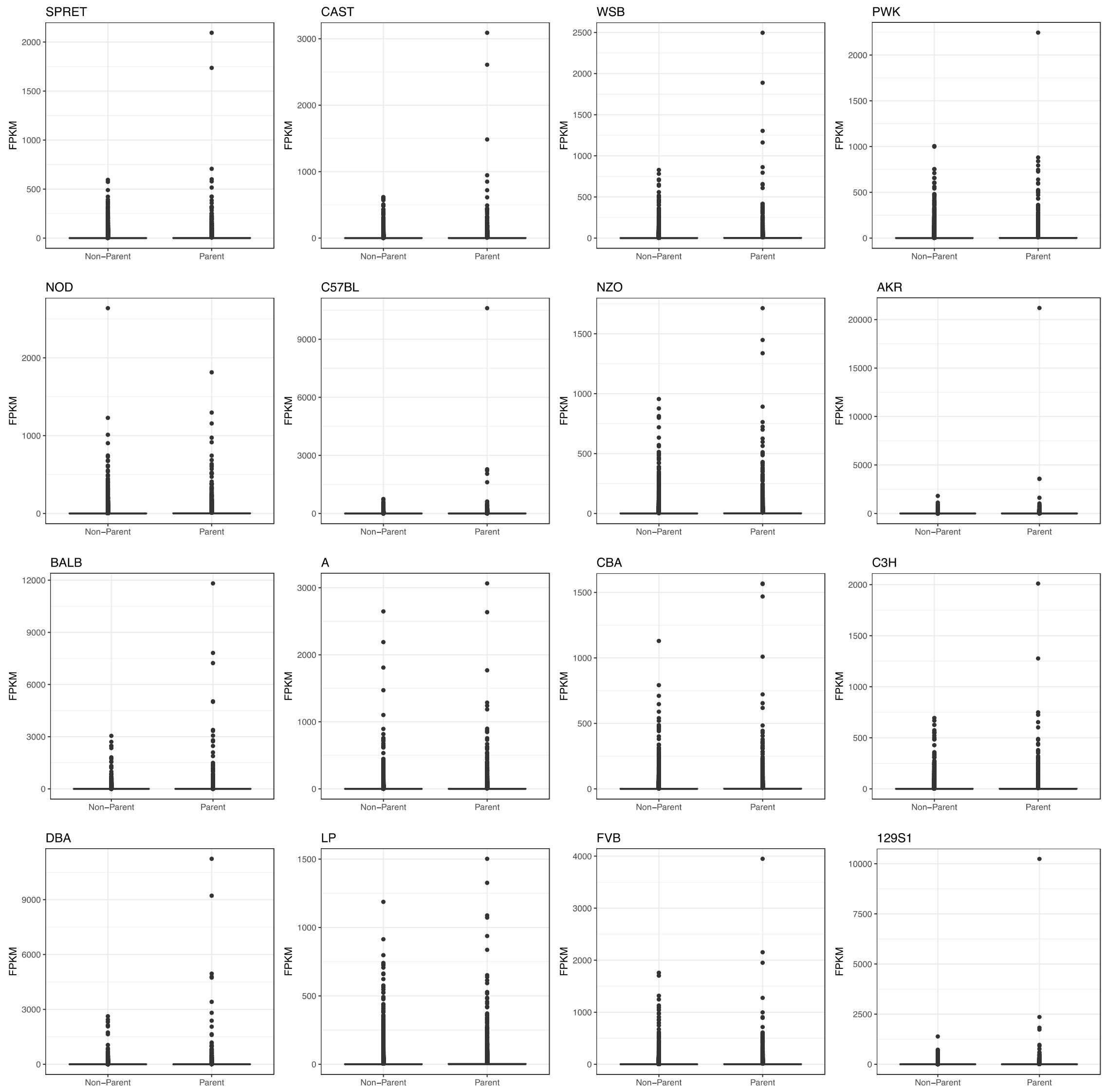

Supplementary Figure 4. D – Average expression levels in adult mouse brain for pseudogene parent and non-parent protein coding genes. The number of samples in each strain is C57BL (parent = 10520, non-parent = 68845), SPRET (parent = 9634, non-parent = 69897), PWK (parent = 9588, non-parent = 70254), CAST (parent = 9706, non-parent = 70441), WSB (parent = 9769, non-parent = 68666), NOD$_{\lambda}$ (parent = 10273, non-parent = 68647), NZO$_{\lambda}$ (parent = 10592, non-parent = 69111), AKR$_{\lambda}$ (parent = 10373, non-parent = 69040), BALB$_{\lambda}$ (parent = 10414, non-parent = 68924), A$_{\lambda}$ (parent = 10386, non-parent = 69019), CBA$_{\lambda}$ (parent = 10240, non-parent = 68833), C3H$_{\lambda}$ (parent = 10250, non-parent = 68863), DBA$_{\lambda}$ (parent = 10300, non-parent = 68870), LP$_{\lambda}$ (parent = 10250, non-parent = 68795), FVB$_{\lambda}$ (parent = 10177, non-parent = 68770), 129S1$_{\lambda}$ (parent = 10226, non-parent = 68933). Centre line indicates the median value, box limits are the upper and lower quartiles, whiskers are 1.5x interquartile range, and the points are the outliers.

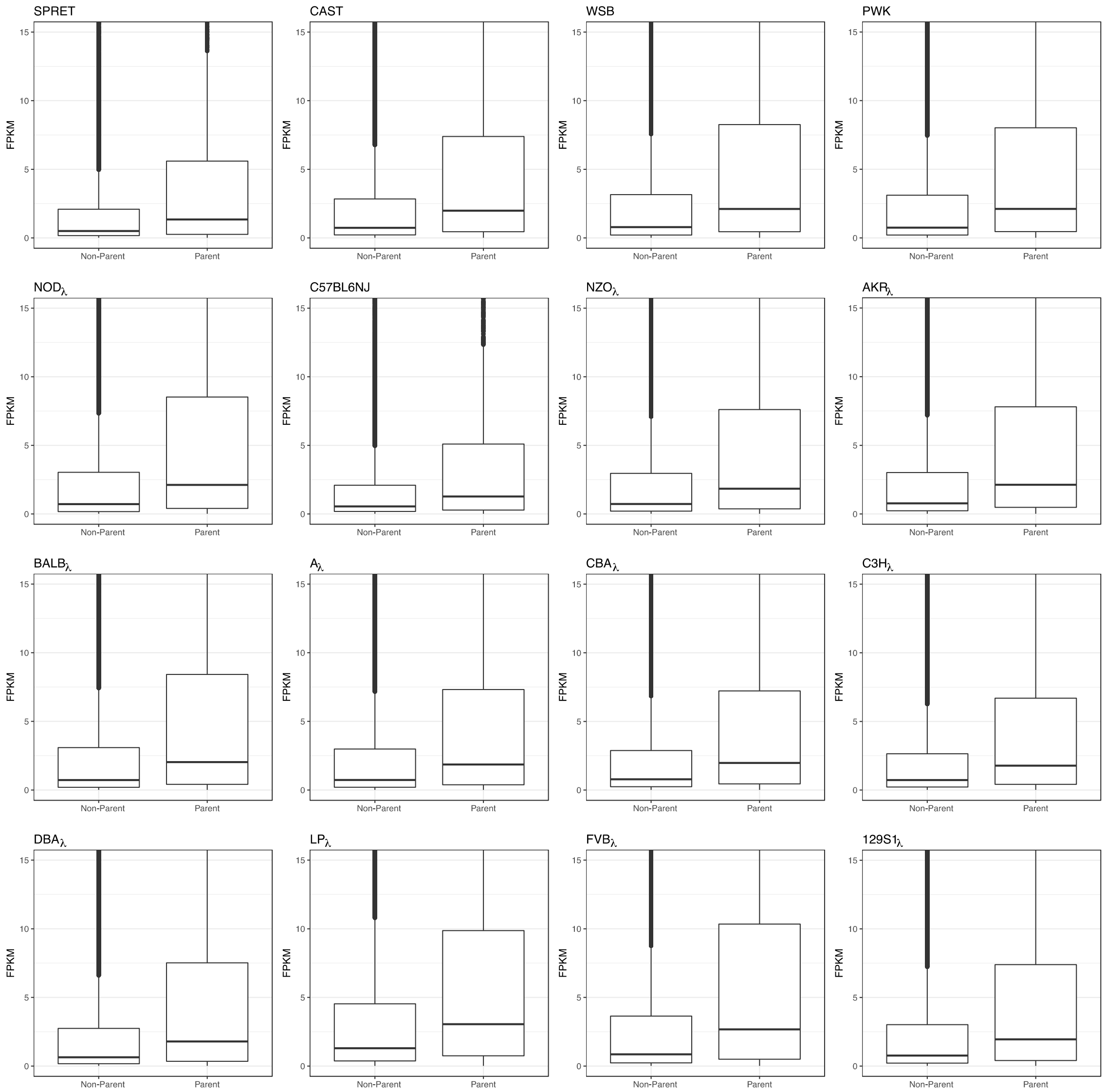

Supplementary Figure 4. E – zoomed in version of **Supplementary Figure 4D**. Average expression levels in adult mouse brain for pseudogene parent and non-parent protein coding genes. Centre line indicates the median value, box limits are the upper and lower quartiles, whiskers are 1.5x interquartile range, and the points are the outliers. The number of samples in each strain is C57BL (parent = 10520, non-parent = 68845), SPRET (parent = 9634, non-parent = 69897), PWK (parent = 9588, non-parent = 70254), CAST (parent = 9706, non-parent = 70441), WSB (parent = 9769, non-parent = 68666), NOD$_{\lambda}$ (parent = 10273, non-parent = 68647), NZO$_{\lambda}$ (parent = 10592, non-parent = 69111), AKR$_{\lambda}$ (parent = 10373, non-parent = 69040), BALB$_{\lambda}$ (parent = 10414, non-parent = 68924), A$_{\lambda}$ (parent = 10386, non-parent = 69019), CBA$_{\lambda}$ (parent = 10240, non-parent = 68833), C3H$_{\lambda}$ (parent = 10250, non-parent = 68863), DBA$_{\lambda}$ (parent = 10300, non-parent = 68870), LP$_{\lambda}$ (parent = 10250, non-parent = 68795), FVB$_{\lambda}$ (parent = 10177, non-parent = 68770), 129S1$_{\lambda}$ (parent = 10226, non-parent = 68933).

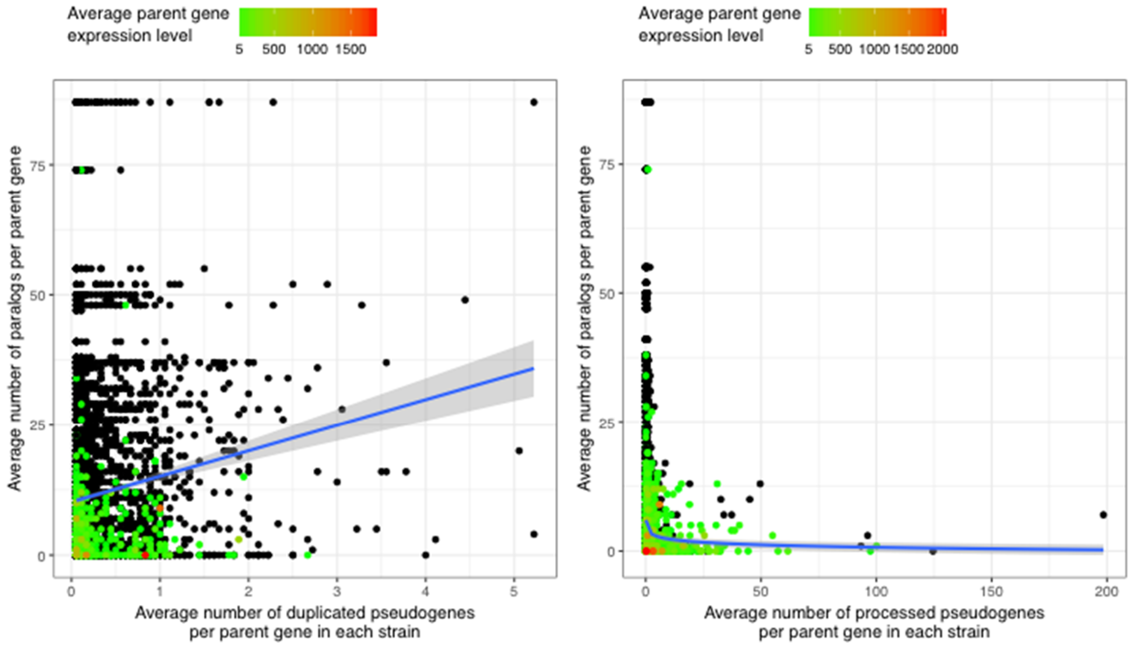

Supplementary Figure 5. A – Relationship between the number of pseudogenes and functional paralogs for a given parent gene (left – duplicated pseudogenes, right – processed pseudogenes). The number of parent genes associated with processed pseudogenes in strains is 11,571, and the number of parent genes associated with duplicated pseudogenes in strains is 3,758. The average number of pseudogenes per parent per strain was obtained by dividing the total number of pseudogenes across all strains by the total number of strains (18). Fitting lines show a vague correlation between the number of functional vs. disabled copies of a gene, with a linear fit for duplicated pseudogenes ($y=4.93x+10.13$) and a negative logarithmic fit ($y=-0.59\log(1/x)+3.99$) for processed pseudogenes. The gray area is the ± SD (standard deviation) of the fitting curve. The dots are coloured by the average expression level of the parent gene in brain adult tissue in the range described in the heat scale above each figure. The black dots correspond to protein coding gene with an average expression level across the strains lower than 5 FPKM.

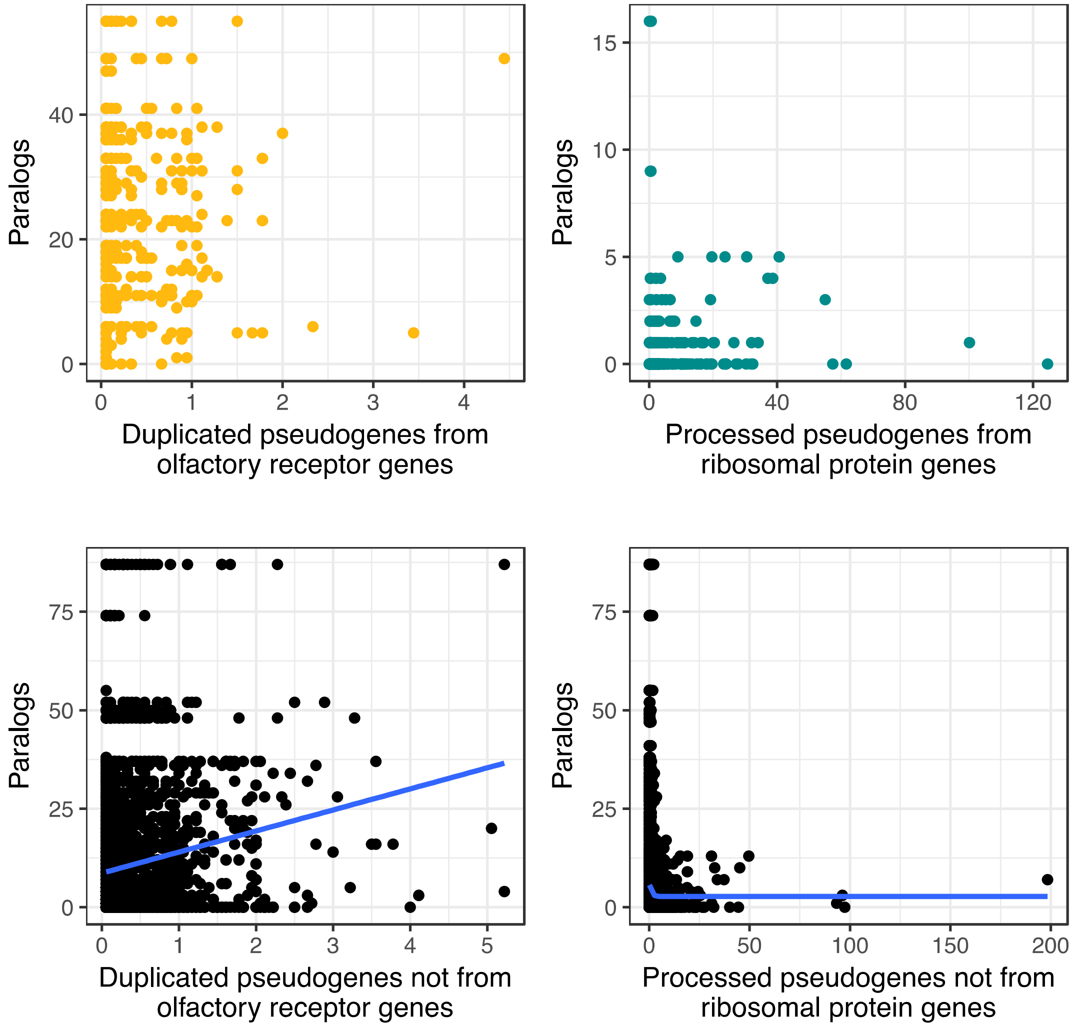

Supplementary Figure 5. B – Relationship between the number of pseudogenes and functional paralogs for a given parent gene (left – duplicated pseudogenes, right – processed pseudogenes) for olfactory receptors (OR) and ribosomal protein (RP) derived pseudogenes. The top left plot shows the distribution of OR pseudogenes vs paralogs of olfactory receptors per strain. Correspondingly, the top right plot shows the distribution of RP pseudogenes vs paralogs of ribosomal proteins per strain. The bottom plots show the distribution of the pseudogenes and paralogs that are not generated from olfactory receptor or ribosomal proteins. Correlation lines are drawn in blue.

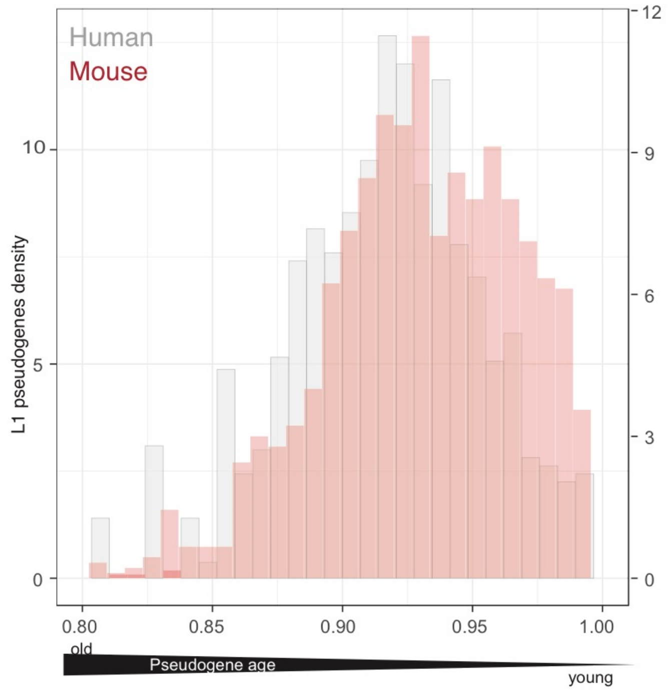

Supplementary Figure 5. C – Distribution of L1-flanked pseudogenes (y-axis) as function of age (x-axis) in human (n=8,081) and mouse (n=9,979). The pseudogene age is approximated as DNA sequence similarity to the parent gene.

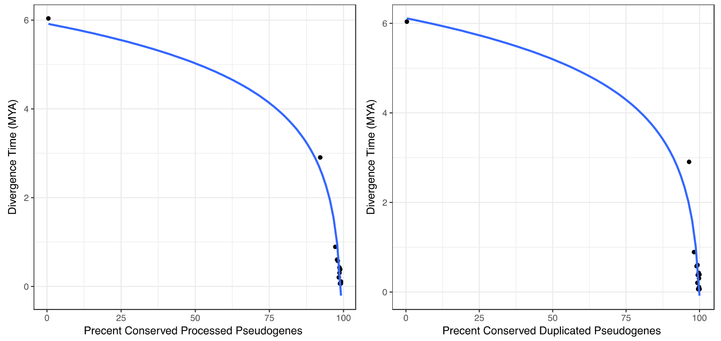

Supplementary Figure 6. Distribution of conserved pseudogenes as function of biotype and strain divergence. The "Misc" biotype includes unitary pseudogenes as well as pseudogene for which the biotype could not be accurately determined. All three pseudogene classes follow a logarithmic curve with respect to the strain divergence times, with the best fit being observed for processed pseudogenes.

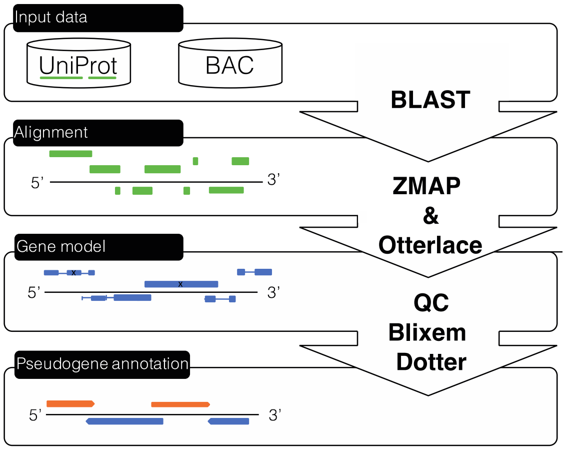

Supplementary Figure 7. Manual annotation curation workflow as previously described in Pei *et al.* (2012).

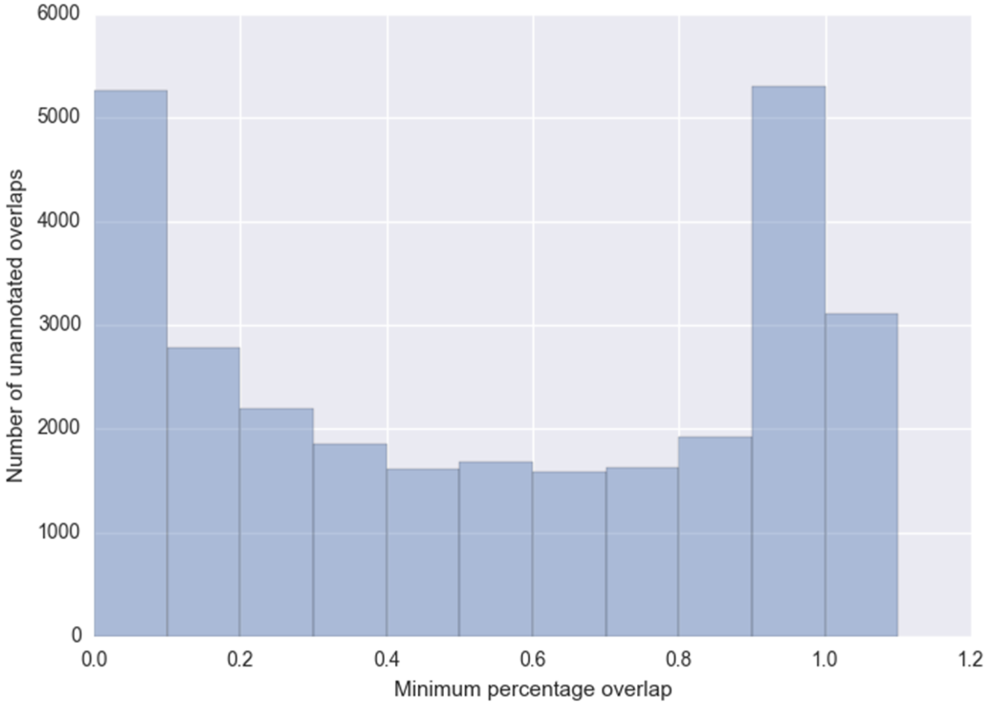

Supplementary Figure 8. Histogram of percentage overlap for lower of the reciprocal overlap cut-offs. Associated data is available from **Supplementary data 8**.

Supplementary Tables

Supplementary Table 1. Reference genome pseudogene annotation in mouse and human.

Organism	Manual curation (M)	PseudoPipe* (PP)	RetroFinder* (RF)	Union PP&RF	Intersection M&PP (%)
**Mouse**	10,524	18,659	18,467	26,103	8,786 (83.5)
**Human**	14,650	15,978	15,474	22,396	13,177 (89.9)

*Chromosomal assembled DNA only

Supplementary Table 2. Reference genome automatic pseudogene annotation in mouse and human.

	**PseudoPipe (PP)**			**RetroFinder (RF)**	**PP-RF overlap**
	Autosomes	Sex Chr.	Others*		
**Mouse**	14,094	4,565	4,162	18,467	10,522
**Human**	14,638	1,341	2,054	15,474	9,057

*Includes patches, scaffolds, and unassembled DNA.

Supplementary Table 3. Human and mouse pseudogene annotation summary.

	**Human (v25)**	**Mouse (M12)**
**Total GENCODE**	**14,650**	**10,524**
processed pseudogenes	10,725	7,486
unprocessed pseudogenes	3,400	2,625
unitary pseudogenes	214	34
polymorphic pseudogenes	51	77
ambiguous pseudogenes	21	99
**Total PseudoPipe**	**15,978 (+2,054*)**	**18,659 (+4,162*)**
processed pseudogenes	8,081 (+ 683*)	9,979 (+ 559*)
unprocessed pseudogenes	2,534 (+ 550*)	1,929 (+ 274*)
ambiguous pseudogenes	5,363 (+ 821*)	6,751 (+3,329*)

*****Includes patches, scaffolds, and unassembled DNA.

Supplementary Table 4. Mouse strains description and nomenclature. The lambda "λ" symbol indicates that a strain is belonging to the classical laboratory inbred strains group.

**Strain ID**	**Description**	**Group**
PAHARI	PAHARI/EiJ – Mus Pahari	Wild-derived outgroup
CAROLI	CAROLI/EiJ – Mus Caroli	
SPRET	SPRET/EiJ – Mus Spretus	
PWK	PWK/PhJ – Mus Musculus Musculus	Wild-derived inbred strains
CAST	CAST/EiJ – Mus Musculus Castaneus	
WSB	WSB/EiJ – Mus Musculus Domesticus	
NOD$_\lambda$	NOD/ShiLtJ – Mus Musculus Non-obese Diabetic	Classical laboratory inbred strains
C57BL	C57BL/6NJ – Mus Musculus Black 6N	
NZO$_\lambda$	NZO/HlLtJ – Mus Musculus New Zealand Obese	
AKR$_\lambda$	AKR/J – Mus Musculus	
BALB$_\lambda$	BALB/cJ – Mus Musculus	
A$_\lambda$	A/J – Mus Musculus	
CBA$_\lambda$	CBA/J – Mus Musculus	
C3H$_\lambda$	C3H/HeJ – Mus Musculus	
DBA$_\lambda$	DBA/2J – Mus Musculus	
LP$_\lambda$	LP/J – Mus Musculus	
FVB$_\lambda$	FVB/NJ – Mus Musculus	
129S1$_\lambda$	129S1/SvImJ – Mus Musculus	

Supplementary Table 5: Estimation of the total number of pseudogenes according to PseudoPipe per strain, the number of pseudogenes in each annotation confidence level, and the number of pseudogenes for each biotype group.

Strain	PseudoPipe predictions	Input protein coding transcripts conserved between reference & strains	%Protein coding transcripts conserved	%Pseudogenes annotated with respect to the total number of pseudogenes in reference genome	Estimate of the total number of PseudoPipe pseudogenes	Level 1	Level 2	Level 3	Processed	Duplicated	Ambiguous	Unitary
**Mouse**	18659	56999	100.00	100.00	**18659**	8786	1738	8135	9980	1930	8487	271
**C57BL/6NJ**	14722	47145	82.71	79.27	**18659**	5615	993	6597	10859	1661	671	14
PAHARI	12414	41022	71.97	68.97	**18082**	2971	1254	6361	9137	1011	426	9
CAROLI	13399	43056	75.54	72.39	**18595**	3860	1224	6362	9640	1295	499	6
SPRET	14170	44567	78.19	74.93	**18998**	4444	980	6511	10137	1242	543	17
PWK	14485	44313	77.74	74.50	**19532**	4630	865	6668	10294	1325	530	15
CAST	14427	45527	79.87	76.55	**18935**	4694	1003	6707	10216	1549	625	15
WSB	14202	46107	80.89	77.52	**18405**	4869	873	6360	10168	1336	584	32
NOD_{λ}	14965	45869	80.47	77.12	**19495**	5285	937	6732	10725	1589	625	11
NZO_{λ}	13909	47417	83.19	79.72	**17527**	5592	1048	6237	10762	1465	637	14
AKR_{λ}	14380	46662	81.86	78.45	**18414**	5289	996	6629	10791	1496	613	6
$BALB_{\lambda}$	14393	46636	81.82	78.41	**18441**	5344	939	6728	10786	1598	613	13
A_{λ}	13823	46760	82.04	78.62	**17664**	5295	997	6448	10684	1417	624	78
CBA_{λ}	14479	46243	81.13	77.75	**18709**	5231	898	6713	10710	1494	624	14
$C3H_{\lambda}$	14400	46360	81.33	77.95	**18560**	5201	917	6618	10665	1455	601	11
DBA_{λ}	13872	46375	81.36	77.97	**17874**	5282	908	6219	10451	1335	609	11
LP_{λ}	13923	46384	81.38	77.99	**17936**	5199	1015	6474	10626	1418	629	13
FVB_{λ}	14202	46205	81.06	77.69	**18366**	5257	977	6460	10652	1430	597	16
$129S1_{\lambda}$	13820	46726	81.98	78.56	**17673**	5284	1042	6501	10616	1591	607	78

Supplementary Table 6: Distribution of numbers of conserved and unconserved pseudogene loci.

Strain	Unconserved	Conserved
PAHARI	4216	442
CAROLI	774	5276
SPRET	239	6338
PWK	202	6572
CAST	221	7068
WSB	178	7343
NOD$_\lambda$	210	8126
NZO$_\lambda$	188	8238
AKR$_\lambda$	161	7966
BALB$_\lambda$	235	8400
A$_\lambda$	176	7942
CBA$_\lambda$	152	8044
C3H$_\lambda$	150	8050
DBA$_\lambda$	159	7914
LP$_\lambda$	142	7950
FVB$_\lambda$	215	7883
129S1$_\lambda$	225	8304

Supplementary Table 7. Enrichment of pseudogene parent gene class in essential genes. The statistical significance was calculated using a two tailed t-test.

Pseudogenes	Genes	Essential	Nonessential	Odds Ratio	p-Value
Total	Parent	1162	1061	1.93	$7.7*10^{-39}$
	Non-Parent	2050	3620		
Processed	Parent	1034	869	2.08	$2.3*10^{-43}$
	Non-Parent	2178	3812		
Duplicated	Parent	334	349	1.44	$6.0*10^{-6}$
	Non-Parent	2878	4332		

Supplementary Table 8. Correlations between gene essentiality and parent gene status controlling for transcription level.

	Linear Prob. Model	Probit	Probit Marginal Effect
Parent gene (Y/N)	0.2035<br>(0.0168)	0.5108<br>(0.0441)	0.1943<br>(0.016)
Transcription	0.0003<br>(0.0001)	0.0010<br>(0.0002)	0.0004<br>(8.11e-05)

Marginal effect for probit (column 3) calculated at mean values for each independent variable. Number of observations: 7,797. Standard errors are given in parentheses. Parent gene (Y/N) is a binary categorical variable that is equal to 1 if a gene has any associated pseudogenes and 0 if not.
