# Supplementary material for: Transcriptional activity and strain-specific history of mouse pseudogenes
Source: Nat Commun. 2020 Jul 29;11:3695. doi: 10.1038/s41467-020-17157-w (PMC7392758; doi:10.1038/s41467-020-17157-w)
Supplement: Supplementary file 2 — Reporting Summary [file 41467_2020_17157_MOESM2_ESM.pdf]

nature research

Corresponding author(s): Mark B Gerstein

Last updated by author(s): May 27, 2020

Reporting Summary

Nature Research wishes to improve the reproducibility of the work that we publish. This form provides structure for consistency and transparency in reporting. For further information on Nature Research policies, see our Editorial Policies and the Editorial Policy Checklist.

Statistics

For all statistical analyses, confirm that the following items are present in the figure legend, table legend, main text, or Methods section.

n/a	Confirmed	
☐	☒	The exact sample size (*n*) for each experimental group/condition, given as a discrete number and unit of measurement
☐	☒	A statement on whether measurements were taken from distinct samples or whether the same sample was measured repeatedly
☐	☒	The statistical test(s) used AND whether they are one- or two-sided<br>*Only common tests should be described solely by name; describe more complex techniques in the Methods section.*
☐	☒	A description of all covariates tested
☐	☒	A description of any assumptions or corrections, such as tests of normality and adjustment for multiple comparisons
☐	☒	A full description of the statistical parameters including central tendency (e.g. means) or other basic estimates (e.g. regression coefficient) AND variation (e.g. standard deviation) or associated estimates of uncertainty (e.g. confidence intervals)
☐	☒	For null hypothesis testing, the test statistic (e.g. *F*, *t*, *r*) with confidence intervals, effect sizes, degrees of freedom and *P* value noted<br>*Give P values as exact values whenever suitable.*
☒	☐	For Bayesian analysis, information on the choice of priors and Markov chain Monte Carlo settings
☒	☐	For hierarchical and complex designs, identification of the appropriate level for tests and full reporting of outcomes
☐	☒	Estimates of effect sizes (e.g. Cohen's *d*, Pearson's *r*), indicating how they were calculated

Our web collection on statistics for biologists contains articles on many of the points above.

Software and code

Policy information about availability of computer code

Data collection	All data is publicly available and no software was used for data collection.
Data analysis	The annotation was performed using PseudoPipe that is publicly available: http://www.pseudogene.org/pseudopipe/, RetroFinder, and BedTools v2.25.0.<br>The liftover of annotations between various strains was done using HAL v2.1 tool halLiftover.<br>The transcription analysis was done using TopHat v2.1.1 and Cuflinks v2.2.1 that are publicly available. Statistical analysis was done using R v 3.3.3 and Python StatsModels package. Mappability maps were created using the GEM library.<br>In the phylogenetic analysis, we randomly selected 1460 out of the 2925 cross strain conserved pseudogenes accounting for about 50% of the entire set, due to software limitations. The software used for the analysis is GENEIOUS 10.2 software package and the aligner used is MUSCLE v3.5. The tree was generated with PhyML 3.0 using the Tamura-Nei genetic distance model and simultaneous Nearest Neighbor Interchange build method.<br>Transposable elements analysis was done using Repbase 21.11 library and RepeatMasker 3.2.8.<br>GO and Pfam analysis was done using R v3.3.3 biomaRt, goSTAG, and OntologyX packages, all that are publicly available.<br>Gene enrichment analysis was conducted using the StatsModels package in Python v2.7.

For manuscripts utilizing custom algorithms or software that are central to the research but not yet described in published literature, software must be made available to editors and reviewers. We strongly encourage code deposition in a community repository (e.g. GitHub). See the Nature Research guidelines for submitting code & software for further information.

Data

Policy information about availability of data

All manuscripts must include a data availability statement. This statement should provide the following information, where applicable:

- Accession codes, unique identifiers, or web links for publicly available datasets
- A list of figures that have associated raw data
- A description of any restrictions on data availability

All data generated and analysed in this work is available at http://mouse.pseudogene.org. The GENCODE manual annotation data used in this study is available at https://www.gencodegenes.org.
The mouse strains assembled genomes from the Mouse Genome Project are available at https://www.sanger.ac.uk/science/data/mouse-genomes-project.
Mouse tissue RNAseq data is available at https://www.ebi.ac.uk/arrayexpress/experiments/E-MTAB-615/samples/.
Mouse development RNAseq data is available on the SRA under Series GSE66582 https://www.ncbi.nlm.nih.gov/sra?term=SRP055882.
Protein families are available at http://xfam.org .

Field-specific reporting

Please select the one below that is the best fit for your research. If you are not sure, read the appropriate sections before making your selection.

- [x] Life sciences
- [ ] Behavioural & social sciences
- [ ] Ecological, evolutionary & environmental sciences

For a reference copy of the document with all sections, see nature.com/documents/nr-reporting-summary-flat.pdf

Life sciences study design

All studies must disclose on these points even when the disclosure is negative.

Sample size	In the phylogenetic analysis, we randomly selected 1460 out of the 2925 cross strain conserved pseudogenes accounting for about 50% of the entire set, due to software limitations. The software used for the analysis is GENEIOUS 10.2 software package and the aligner used is MUSCLE v3.5. The tree was generated with PhyML 3.0 using the Tamura-Nei genetic distance model and simultaneous Nearest Neighbor Interchange build method.<br>In all other analyses, all available data was used.
Data exclusions	No data was excluded from the analysis.
Replication	Not applicable. All the results presented in this work are based on publicly available data from large scale sequencing projects. No additional experiments were designed or conducted, and no replicates were used.
Randomization	The random selection of the pseudogenes in phylogenetic analysis was performed using the random.seed() function in Python 2.7. For all the other experiments all available data was used and no randomization was performed. The data was grouped based on the organism of origin.
Blinding	Not applicable.

Reporting for specific materials, systems and methods

We require information from authors about some types of materials, experimental systems and methods used in many studies. Here, indicate whether each material, system or method listed is relevant to your study. If you are not sure if a list item applies to your research, read the appropriate section before selecting a response.

Materials & experimental systems

n/a	Involved in the study	
x		Antibodies
x		Eukaryotic cell lines
x		Palaeontology and archaeology
x		Animals and other organisms
x		Human research participants
x		Clinical data
x		Dual use research of concern

Methods

n/a	Involved in the study	
x		ChIP-seq
x		Flow cytometry
x		MRI-based neuroimaging
