# Supplementary material for: Transcriptional activity and strain-specific history of mouse pseudogenes
Source: Nat Commun. 2020 Jul 29;11:3695. doi: 10.1038/s41467-020-17157-w (PMC7392758; doi:10.1038/s41467-020-17157-w)
Supplement: Supplementary file 3 — Description of Additional Supplementary Files [file 41467_2020_17157_MOESM3_ESM.pdf]

Description of Additional Supplementary Files

File Name: Supplementary Data 1
Description: Unitary pseudogenes in human and mouse. (SupData.1.xlsx also available at http://mouse.pseudogene.org/Supplement/)

File Name: Supplementary Data 2
Description: Pseudogene family and clan characterization. (SupData.2.xlsx also available at http://mouse.pseudogene.org/Supplement/)

File Name: Supplementary Data 3
Description: Unitary pseudogenes in mouse strains. (SupData.3.xlsx also available at http://mouse.pseudogene.org/Supplement/)

File Name: Supplementary Data 4
Description: Encode transcription data. (SupData.4.xlsx also available at http://mouse.pseudogene.org/Supplement/)

File Name: Supplementary Data 5
Description: Matrix of enrichment values for Gene Ontology terms in the 18 mouse strains. (SupData.5.xlsx also available at http://mouse.pseudogene.org/Supplement/)

File Name: Supplementary Data 6
Description: Transcribed pseudogenes in mouse reference genome. (SupData.6.xlsx also available at http://mouse.pseudogene.org/Supplement/)

File Name: Supplementary Data 7
Description: Pangenome pseudogene functional annotation and characterisation dataset. (SupData.7.xlsx also available at http://mouse.pseudogene.org/Supplement/)

File Name: Supplementary Data 8
Description: Mouse strains haplotype data. (SupData.8.xlsx also available at http://mouse.pseudogene.org/Supplement/)
